# Mapping the transcriptional diversity of calcium signaling in the mouse and human brain

**DOI:** 10.1016/j.isci.2026.116055

**Published:** 2026-05-28

**Authors:** Ibrahim Al Rayyes, Lauri Louhivuori, Ivar Dehnisch Ellström, Erik Smedler, Per Uhlén

**Affiliations:** 1 Division of Molecular Neurobiology, Department of Medical Biochemistry and Biophysics, Karolinska Institutet, 17177 Stockholm, Sweden; 2 Department of Neuroscience and Physiology, University of Gothenburg, 40530 Gothenburg, Sweden; 3 The Wallenberg Centre for Molecular and Translational Medicine, 40530 Gothenburg, Sweden; 4 Psykiatri Affektiva, Department of Psychiatry, Region Västra Götaland, 41132 Gothenburg, Sweden

**Keywords:** Biological sciences, Neuroscience, Transcriptomics

## Abstract

Calcium (Ca^2+^) signaling is a key regulator of brain function and development. Here, we comprehensively analyze the Ca^2+^ signaling transcriptome in the adult mouse brain and the developing human brain to reveal the basis of signaling specificity. We show that neurons organize into non-stochastic Ca^2+^ states that reflect cell-type identity and capture subtle functional differences. These states arise from lineage-specific developmental Ca^2+^ programs that are detectable already in progenitor stages, and may precede differentiation into mature neuronal cell types. During neocortical development, many Ca^2+^ signaling genes, such as *ADGRV1, NCALD,* and *CREB5*, peak at distinct developmental stages, are evolutionarily conserved, and reflect transcriptional heterogeneity within progenitors associated with cell-fate decisions. Together, our findings provide an in-depth understanding of how a tightly regulated Ca^2+^ signaling transcriptome encodes cell-state-specific signaling programs and demonstrate that Ca^2+^ signaling is precisely tailored to distinct cell states.

## Highlights

• Neurons organize into distinct, non-stochastic Ca^2+^ states
• Ca^2+^ states reflect cell identity and subtle functional differences
• Ca^2+^ signaling diversity emerges from lineage-specific developmental programs
• Neocortical Ca^2+^ genes are tightly regulated and evolutionarily conserved

## Graphical abstract

**Figure:**
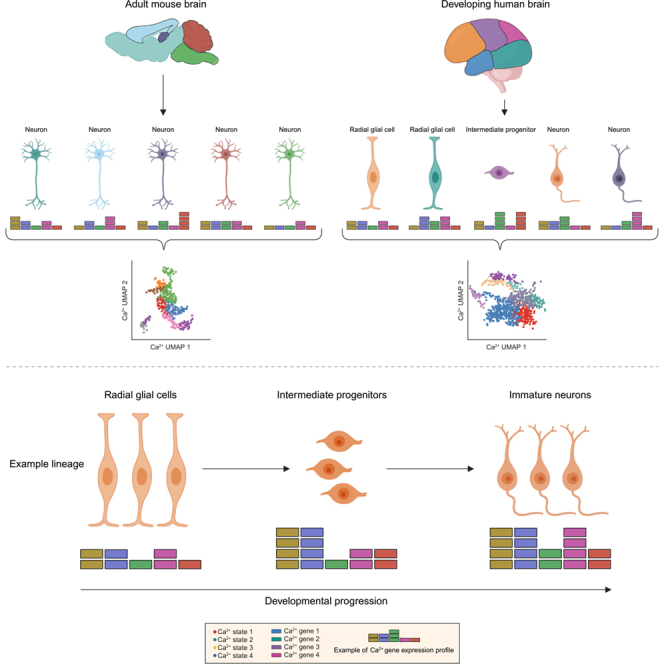

## Introduction

Calcium ions (Ca^2+^) are versatile intracellular signaling messengers that regulate most neuronal functions, including synaptic plasticity, neurotransmitter release, excitability, and activity-dependent gene transcription.^1^^,^^2^^,^^3^ Through interactions with developmentally important signaling pathways, such as Notch and Wnt signaling,^4^^,^^5^ morphogens^6^ and transcription factors (TFs),^7^ Ca^2+^ signaling is known to regulate nearly every aspect of neurogenesis, synaptogenesis, and experience-dependent plasticity across multiple regions, including the neocortex, cerebellum, dentate gyrus (DG), and olfactory (OL) bulb.^8^^,^^9^ This versatility is enabled by the Ca^2+^ signaling protein toolkit, a broad repertoire of proteins that control, transduce, and execute Ca^2+^ signals to elicit downstream effects. Given the diversity in neuronal functions, firing properties, and connectivity, as well as the complex spatiotemporal dynamics of morphogens and patterning genes that ultimately guide developmental programs across the brain, the involvement of Ca^2+^ signaling in these processes raises the question of how different outputs from Ca^2+^ signals are achieved.

One hypothesis suggests that the tissue-specific expression of subsets of genes that encode the Ca^2+^ signaling protein toolkit, here referred to as the Ca^2+^ signaling transcriptome, could enable cells to achieve and maintain functional specificity.^1^^,^^10^ Whether this hypothesis applies to the brain remains an unaddressed question. This is further complicated by the neuronal activity-dependent regulation of many Ca^2+^ genes, which raises the question of the extent to which the expression of Ca^2+^ genes reflects differences in functions and activity. Nevertheless, there are examples, such as the use of *PVALB* and *CALB2* to identify specific sub-populations of midbrain dopaminergic and cortical GABAergic neurons,^11^^,^^12^ as well as differences between the cerebellum and DG in how Ca^2+^-mediated mechanisms drive long-term potentiation and depression,^13^ that suggest that the expression of Ca^2+^ genes is, at least partially, independent of differences in activity and raises the possibility that there may be a general pattern of cell- and region-specific transcriptional encoding of Ca^2+^ signaling programs. Understanding the molecular principles underlying the specificity of Ca^2+^ signaling could have significant implications for our comprehension of Ca^2+^-driven processes, including the regulation of neurogenesis across different brain regions.

Until recently, technological limitations have constrained comprehensive analyses of the Ca^2+^ signaling transcriptome, with studies largely restricted to the simultaneous analysis of a few Ca^2+^ genes, resulting in a limited understanding of its large-scale diversity. With the emergence of single-cell RNA sequencing (scRNA-seq), an unprecedented capacity for studying the temporal and cellular heterogeneity of Ca^2+^ signaling in the human and mouse brain in a high-throughput and unbiased manner now exists. Here, we leverage scRNA-seq to systematically map the Ca^2+^ signaling transcriptome across the mouse nervous system and the developing human brain. Our analysis reveals that coordinated combinations of Ca^2+^-regulatory genes define discrete cellular Ca^2+^ states that align with lineage progression, developmental transitions, and functional maturation, suggesting that neurons are transcriptionally primed for specific modes of Ca^2+^ signaling.

## Results

### scRNA-seq reveals an architecture of distinct Ca^2+^-states across the nervous system

**Figure 1:**
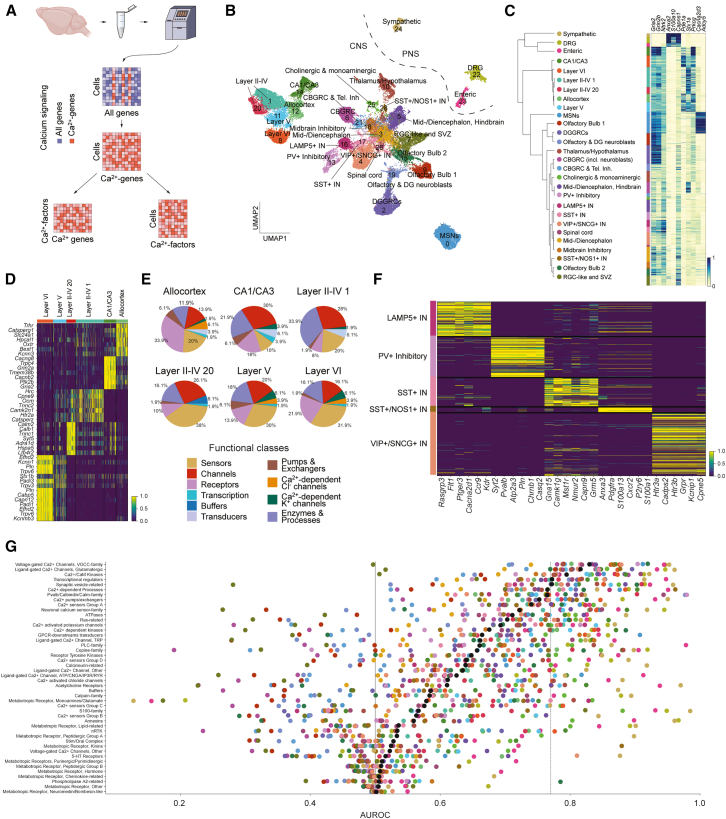
Characterization of single-cell Ca^2+^-states across the mouse nervous system (A) Schematic overview of the analysis workflow. All non-Ca^2+^-genes were excluded, and the remaining unique molecular identifier (UMI) counts were decomposed using single-cell Hierarchical Poisson Factorization (*scHPF*). The resulting gene- and cell-factor scores were used for downstream clustering and gene expression analyses. (B) UMAP embedding based on Ca^2+^-genes of 63,507 neurons from Zeisel et al.,^14^ clustered into 28 distinct Ca^2+^-states. (C) *Left:* Dendrogram constructed from the high-dimensional UMAP representation, illustrating the hierarchical relationships among Ca^2+^-states. *Right:* Heatmap showing representative genes associated with successive splits across the mouse nervous system. Rows represent individual cells, grouped by Ca^2+^-states. (D) Heatmap of Ca^2+^-gene enrichment across telencephalic excitatory neuronal Ca^2+^-states. (E) Pie charts show the distribution of 10 broad Ca^2+^ functional classes, derived by grouping related functional groups, among the top 50 Ca^2+^-genes for each telencephalic excitatory neuronal Ca^2+^-state. (F) As in (D), but for telencephalic interneuron Ca^2+^-states. (G) Scatterplot shows area under the receiver operating characteristic curve (AUROC) values for all 46 Ca^2+^ functional groups, ordered by mean performance. Colors indicate Ca^2+^-states from (B).

To gain a comprehensive understanding of the Ca^2+^ signaling transcriptome in the nervous system, we curated a list of Ca^2+^ signaling genes (hereafter referred to as Ca^2+^-genes; see terminology conventions and STAR Methods) and analyzed their expression in the adult mouse nervous system using data from Zeisel et al.,^14^ totaling 133 sample brains from P12-P30. We applied single-cell hierarchical Poisson factorization (scHPF)^15^ to decompose expression counts of the Ca^2+^-genes into 27 latent factors (hereafter referred to as Ca^2+^-factors; Figures 1A and S1A–S1C), yielding factor scores for each cell and each gene. We next devised an iterative clustering strategy that utilized the cell-factor scores, identifying 28 distinct Ca^2+^ clusters (hereafter referred to as Ca^2+^-states), which largely reflected the underlying cell-type architecture (Figure 1B; Table S1; STAR Methods), with each Ca^2+^-state enriched for at least one Ca^2+^-factor (Figures S1E and S2). To evaluate the robustness of this approach, assess potential technical batch effects, and determine whether the Ca^2+^-states represented biologically meaningful clusters rather than artifacts of general gene expression differences across cell types, we employed a two-pronged strategy. First, we performed multiple iterations using an equivalent number of randomly selected genes. Second, we trained random forest classifiers to predict both Ca^2+^-states and cell types. Random gene sets failed to recapitulate the Ca^2+^-driven clustering, whereas Ca^2+^-based classifiers robustly predicted both Ca^2+^-states and cell types (Figure S3), indicating that the observed Ca^2+^-driven organization does not simply reflect arbitrary transcriptional differences among cell types.

To link the Ca^2+^-states directly to genes, we derived corrected cell-gene scores from the cell-factor and gene-factor scores (Table S2; Figure S4A; STAR Methods). A dendrogram constructed from the high-dimensional uniform manifold approximation and projection (UMAP) embedding showed that the first split in Ca^2+^-states was between the central nervous system (CNS) and peripheral nervous system (PNS) neurons, marked by differences in genes such as *Gria2* and *Grin2b* or *Anxa2* and *S100a10* (Figure 1C). This was followed by a split separating the telencephalic excitatory neurons, marked by *Stx1a* and *Prkcg*, from medium spiny neurons (MSNs), which were enriched for *Cacna2d3* and *Adcy5,* respectively. The telencephalic excitatory neurons formed six Ca^2+^-states, divided into neocortical layers II-IV, V, and VI, allocortex, and hippocampal CA1/CA3 neurons. Analysis of the top differentially enriched genes showed that each Ca^2+^-state exhibited a distinct molecular signature at both the individual gene level (Figure 1D) and across functional classes (Figure 1E), with particularly pronounced differences in the relative representation of receptors (e.g., *Adra1d* and *Oxtr*), sensors (e.g., *Calb1* and *Calm2*), channels (e.g., *Grin2b* and *Gria1*), and enzymes & processes (e.g., *Camk2n1* and *Capn9*).

Moreover, we found that the telencephalic interneurons formed distinctive Ca^2+^-states that largely adhered to the major interneuron cell types (Figure S4B), with one Ca^2+^-state shared by the VIP+ and SNCG+ interneurons (Figure S4C). Notably, the separation was driven by differences in a range of Ca^2+^-genes, including *Cacna2d1*, *Rasgrp3, Atp2a3, Pln, Camk1g*, *Anxa3*, *S100a13*, *Htr3a*, and *Kcnip1,* illustrating that the differential expression of Ca^2+^-genes in interneurons extends beyond *Pvalb* and *Calb2,* which have commonly been used to classify interneurons (Figure 1F). Notably, the PV+ inhibitory Ca^2+^-state also included non-telencephalic inhibitory neurons, such as the cerebellar (CB) molecular layer interneurons. Additionally, one of the two SST+ Ca^2+^-states was shared with the dopaminergic OL interneurons. Overall, our results extend the established notion of individual Ca^2+^-genes marking certain populations of interneurons by illustrating that subsets of Ca^2+^-genes are differentially enriched across interneurons, with some overlap between cell types across different developmental origins. The fast-spiking nature of both telencephalic PV+ interneurons and PV+ CB molecular layer interneurons^16^^,^^17^ implies that these states may reflect firing properties.

We then categorized the Ca^2+^-genes based on their canonical functions (Table S3) and evaluated how each functional group contributed to the overall transcriptomic heterogeneity in the dataset using *MetaNeighbor*.^18^ First, we pooled the Ca^2+^-states into five groups, partly based on the splits described above: PNS; cortical excitatory neurons; cortical inhibitory interneurons; MSN, and the rest. Across the entire dataset, we found that Ca^2+^-genes belonging to the “voltage-gated Ca^2+^ channels, VGCC-family,” “ligand-gated Ca^2+^ channels, glutamatergic,” “Ca^2+^/CaM kinases,” “Ca^2+^-dependent processes,” “transcriptional regulators,” and “synaptic vesicle-related” groups contributed highly, indicating that these sets are differentially enriched across the Ca^2+^-states. Furthermore, we found that numerous functional groups, including the “S100-family” and “annexins,” performed strongly in predicting the PNS Ca^2+^-states despite a lower overall performance (Figure 1G), suggesting that they were less discernible within the CNS, and supporting that the largest axis of variation in the Ca^2+^ signaling transcriptome was between the CNS and PNS. Together, our results illustrate, for the first time, that Ca^2+^-genes are heterogeneously expressed across the brain in a function- and cell type-dependent manner, raising the possibility that neurons are transcriptionally primed toward specific cell responses or functions.

### The cellular Ca^2+^-states capture activity-driven developmental changes within cortical cell types

**Figure 2:**
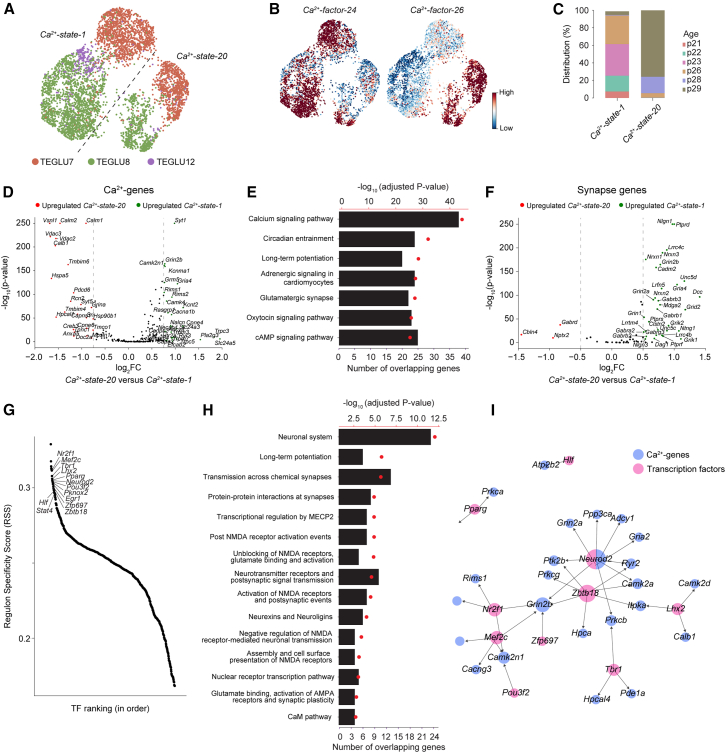
Ca^2+^-states capture activity-driven functional heterogeneity within neocortical cell types (A) UMAP embedding shows neocortical cell types present in *Ca*^*2+*^*-state-1* and *Ca*^*2+*^*-state-20*, indicated by color. The dashed line delineates the boundary between the two Ca^2+^-states. (B) UMAP feature plots show the differential enrichment of *Ca*^*2+*^*-factor-24* and *Ca*^*2+*^*-factor-26*. (C) Stacked bar plots show the age distributions of cells within *Ca*^*2+*^*-state-1* and *Ca*^*2+*^*-state-20*. (D) Volcano plot of differentially expressed Ca^2+^-genes across *Ca*^*2+*^*-state-1* and *Ca*^*2+*^*-state-20*. (E) KEGG pathway enrichment analysis of Ca^2+^-genes upregulated in *Ca*^*2+*^*-state-1* relative to *Ca*^*2+*^*-state-20*, ranked by *p* value. (F) As in (D), but for synapse formation and remodeling-related genes adapted from Südhof.^19^ (G) Top *Ca*^*2+*^*-state-1*-specific TFs, ranked by regulon specificity score (RSS) across the entire mouse dataset. (H) Reactome pathway enrichment analysis of regulons enriched in *Ca*^*2+*^*-state-1* compared with *Ca*^*2+*^*-state-20.* (I) Network representation of TFs predicted to directly regulate Ca^2+^-genes.

Given the diverse roles of Ca^2+^ signaling, we next asked whether Ca^2+^-states derived from the Ca^2+^ signaling transcriptome convey information about neuronal functionality or activity that is independent of taxonomic identity. To address this question, we focused on *Ca*^*2+*^*-state-1* and *Ca*^*2+*^*-state-20,* given that they were predominantly composed of the same cell types from cortical layer II-IV: *TEGLU7, TEGLU8*, and *TEGLU12* (Figure 2A). First, we confirmed that the cells were sourced from multiple donors sequenced across several experiments (Figure S5A), thus making it less likely that the differences in Ca^2+^-factor enrichment (Figure 2B) were caused by batch effects. Next, we confirmed that there were no apparent differences in the expressions of cell type markers between *Ca*^*2+*^*-state-1* and *Ca*^*2+*^*-state-20* (Figure S5B). To further understand the discrepancy between the Ca^2+^-states and the originally defined taxonomy, we analyzed the relationship between Ca^2+^-states and the global transcriptome. Notably, a linear discriminant analysis of the principal components (PCs) of the global transcriptome revealed that the cells adhered to their Ca^2+^-states based on a few PCs (Figure S5C). As the number of PCs increased, a transition toward their taxonomic cell types was observed (Figure S5D), thereby masking the Ca^2+^-states and illustrating that the Ca^2+^-states reflect subtle differences within cell types. In particular, we found that cells in *Ca*^*2+*^*-state-1* were primarily aged P21-P26, whereas those in *Ca*^*2+*^*-state-20* were predominantly P29 (Figure 2C). Differential expression analysis of the Ca^2+^-genes showed that *Ca*^*2+*^*-state-1* exhibited an upregulation of the voltage-gated calcium channel (VGCC) family (e.g., *Cacna1a*), N-methyl-D-aspartate receptor (NMDAR) (e.g., *Grin2b*), and Ca^2+^/Calmodulin kinase II (CAMKII)-related genes (e.g., *Camk2n1*), among others (Figures 2D and S5E; Table S4). Furthermore, gene ontology analysis indicated an overall enrichment for Ca^2+^-genes belonging to “long-term potentiation,” “glutamatergic synapse,” and “cAMP signaling pathway” (Figure 2E; Table S5).

Given that the P21-P26 align with the “critical period,” a phase marked by heightened potential for experience-driven synaptic plasticity and maturation during postnatal development,^20^ these findings suggest that the division into two Ca^2+^-states might reflect variations in activity-dependent synaptic plasticity. Consistent with this, differential expression analysis of genes known to drive synapse formation and remodeling, adapted from Südhof^19^ (Table S6), revealed that many were upregulated in *Ca*^*2+*^*-state-1* (Figure 2F), including Neurexins (*Nrxn1*, *Nrxn2, Nrxn3*), Neuroligins (*Nlgn1, Nlgn2*, *Nlgn3*), SynCAM2 (*Cadm2*), LAR-type receptor tyrosine phosphatases (*Ptprs*, *Ptprd, Ptprf*), and GluD2 (*Grid2*). Stratifying by cell type preserved the observed differences between *Ca*^*2+*^*-state-1* and *Ca*^*2+*^*-state-20* (Figure S5F). Additionally, we found that these expression differences were consistently replicated across other neocortical cell types exhibiting similar age distributions (Figure S5G).

Finally, to further validate our findings, we explored whether the observed transcriptional differences between *Ca*^*2+*^*-state-1* and *Ca*^*2+*^*-state-20* were reflected in differences in TF activities. As expected, we found that the predicted TF activities of the two Ca^2+^-states were highly correlated (Spearman correlation = 0.82, Figure S6A), reflecting their overall similarities. Next, we calculated specificity scores for the two Ca^2+^-states across the entire dataset. Among the top 20 TFs, seven were shared, including the upper-layer markers *Emx1* and *Cux1* (Figure S6B). Furthermore, *Ca*^*2+*^*-state-1* was highly specific for *Mef2c*, *Nr2f1*, and *Zbtb18*, among others (Figure 2G), which have been heavily implicated in regulating activity-dependent synapse formation and maturation.^21^^,^^22^^,^^23^ Moreover, gene ontology analysis of the thirteen non-shared TFs and their inferred targets revealed a strong convergence on pathways involved in synaptic plasticity and remodeling (Figures 2H, S6C, and S6D), mediated through coordinated regulation of Ca^2+^-dependent signaling components, including the upregulation of Ca^2+^-genes such as *Camk2n1*, *Camk2a*, and *Grin2b* (Figure 2I). Together, these findings suggest the activation of transcriptional programs in *Ca*^*2+*^*-state-1* that couple genes associated with Ca^2+^ signaling to synaptic strengthening and remodeling, thereby providing a plausible mechanistic link between this Ca^2+^-state and activity-dependent synaptic modification. These results thus exemplify the potential of Ca^2+^-gene-driven analyses to provide deeper biological insights, revealing subtle yet significant differences among highly similar neurons that may otherwise be masked by overall transcriptomic similarities.

### Postnatal remodeling of cellular Ca^2+^-states follows lineage-specific neurogenesis trajectories

**Figure 3:**
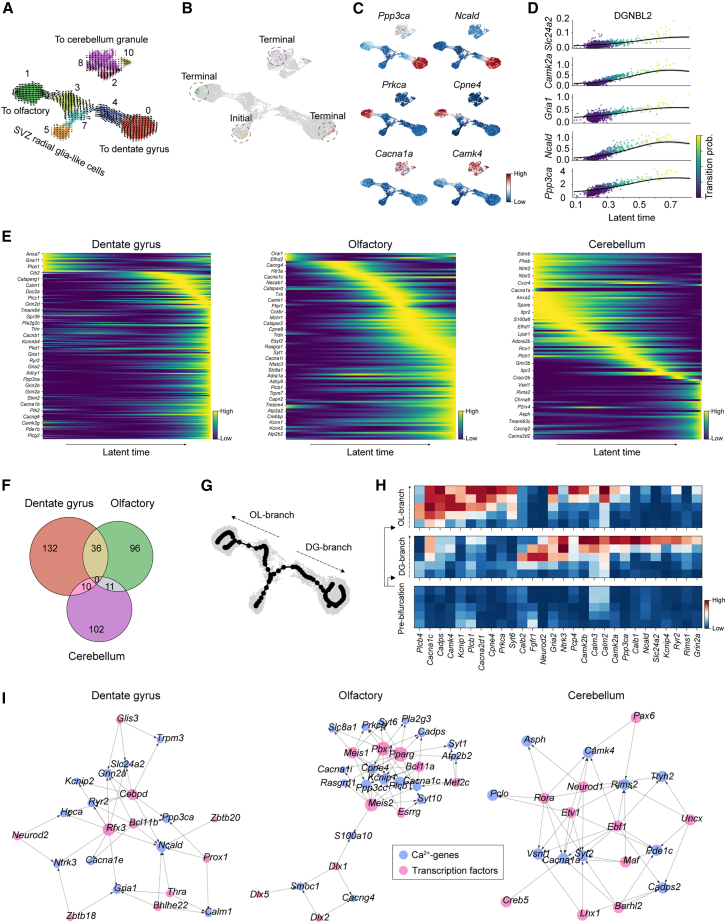
Postnatal remodeling of Ca^2+^-states follows lineage-specific neurogenesis trajectories (A) UMAP embedding shows divergent developmental trajectories based on transcriptomic variation in Ca^2+^-genes. Arrows indicate Ca^2+^-based RNA velocity vectors. (B) Lineage inference using CellRank^30^ based on Ca^2+^-genes identifies immature RG-like cells as the earliest state and three terminal states corresponding to DGGRCs, OL, and CBGRCs. (C) UMAP feature plots show the expression of the Ca^2+^-genes most strongly correlated with each of the three lineages. (D) Scatterplot shows a gradient of increasing expression of terminal dentate gyrus Ca^2+^-genes within the DGNBL2 population. Colors indicate absorption probability. (E) Expression dynamics of Ca^2+^-genes along latent time toward each lineage endpoint, revealing large-scale, lineage-specific transcriptional changes. (F) Venn diagram shows the overlap of Ca^2+^-genes between the three lineages. (G) Principal tree inferred using scFates capturing bifurcation of Ca^2+^-genes between the dentate gyrus (DG) and olfactory (OL) lineages. (H) Heatmap shows genes upregulated in the OL branch (top) and DG branch (middle), before and after the bifurcation point. (I) TF-Ca^2+^-gene regulatory networks show lineage-specific TFs predicted to directly regulate lineage-specific Ca^2+^-genes across the three trajectories.

Notably, the DG and OL radial glial cell (RGC)-like cells formed a common Ca^2+^-state together with the subventricular zone progenitors. Furthermore, the UMAP embeddings showed that this Ca^2+^-state bordered the DG and OL neuroblasts, which in turn were juxtaposed with the DG granule neurons and OL neurons (Figure 1B), mimicking lineage trajectories. The increasing divergence with maturation warranted a closer analysis of the dynamics of the Ca^2+^-states during postnatal development. Accordingly, we subset the dataset to include all neural progenitors, neuroblasts, and their corresponding neurons. Within this subset, we identified three well-represented cell populations: DG granule neurons, OL inhibitory neurons, and CB granule neurons. We then re-iterated the Ca^2+^-driven clustering on this subset, which refined the separation of the progenitor Ca^2+^-state (Figure S7A) and aligned the cells along three lineage trajectories (Figure 3A), each exhibiting differential enrichment of Ca^2+^-factors (Figure S7B). Next, we performed RNA velocity and cell fate analyses exclusively on the Ca^2+^-genes to contrast their dynamics within and between trajectories. The three types of neurons appeared as terminal Ca^2+^-states, and the early RG-like cells were the only initial Ca^2+^-state (Figures 3B and S7C). Each terminal Ca^2+^-state correlated with different Ca^2+^-genes, such as *Ncald* and *Ppp3ca* (DG), *Prkca* and *Cpne4* (OL), *Camk4* and *Cacna1a* (CB), whereas the RG-like Ca^2+^-state correlated with *Ntsr2, Sparc, Notch1*, *Ednrb,* and several S100A members (Figures 3C, S7D, and S7E). Importantly, correlating TFs with the Ca^2+^-defined trajectories (Figure S7F) captured several TFs previously implicated in neurogenesis and lineage specification, such as *Rfx3, Bcl11b*, and *Prox1* in the DG,^24^^,^^25^^,^^26^ *Meis2* and *Pbx1* in the OL bulb,^27^^,^^28^ and *Etv1* in the CB,^29^ indicating that the Ca^2+^-genes accurately reconstructed the lineage trajectories.

Using the DG trajectory as an example, we noted a gradual increase in the expression of terminal genes within the immature *DGNBL2* population that correlated with pseudotime (Figure 3D), highlighting the potential existence of a gradient of functional maturity in developing neurons. In addition, ordering the lineage-related genes by their pseudotemporal kinetics revealed that the global transcriptional Ca^2+^ dynamics differed between the three trajectories (Figure 3E). As expected from the UMAP embeddings, the DG and OL lineages had many overlapping genes compared to the CB (36 versus 10 and 11, respectively), and no Ca^2+^-genes were shared across all three (Figure 3F). Bifurcation analysis showed that a total of 28 genes drove the bifurcation between the DG and OL lineages, including members of the KCNIP-family of Ca^2+^-dependent TFs (*Kcnip1* and *Kcnip4*), *Camk2a, Camk2b,* and the VGCCs, which were shared between the two branches (Figures 3G and 3H), suggesting that Ca^2+^-genes are significantly influential in controlling the determination of cellular fate. To further examine the regulatory links between Ca^2+^ signaling and the DG and OL lineages, we assessed whether Ca^2+^-genes were among the inferred targets of the top lineage-correlating TFs. We found that these TFs converged on Ca^2+^-genes that exhibited lineage-specificity, such as *Ncald*, *Kcnip1*, and *Camk4* (Figure 3I). Collectively, our results suggest that the Ca^2+^ signaling transcriptome becomes increasingly lineage-specific during postnatal development, likely driven by the direct upregulation of distinct sets of Ca^2+^-genes by key lineage-associated TFs.

### Regional and functional heterogeneity of the Ca^2+^ signaling transcriptome during human embryonic neurogenesis

**Figure 4:**
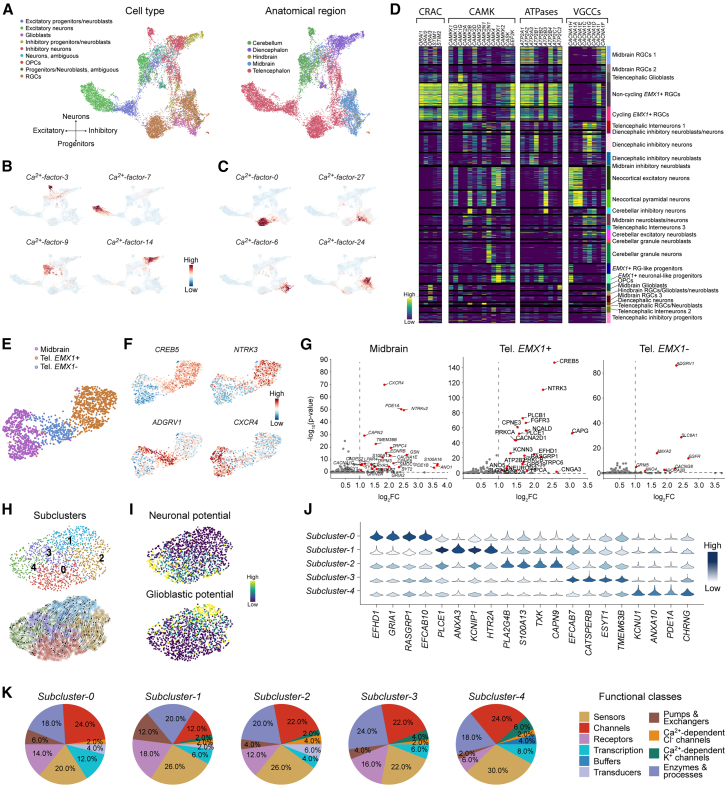
Transcriptional Ca^2+^ dynamics during early human brain development (A) UMAP embedding based on Ca^2+^-genes of data from van Bruggen et al.,^31^ colored by cell type (left) and anatomical region (right). (B) UMAP feature plots illustrate examples of neuronal cell-type-specific Ca^2+^-factors. (C) UMAP feature plots highlight the regional specificity of Ca^2+^-factors within progenitor populations. (D) Heatmap shows the differential enrichment of individual members of canonical Ca^2+^ signaling pathways. From left to right: Ca^2+^ release-activated channels (CRACs), Ca^2+^/calmodulin kinases (CAMKs), ATPases, and voltage-gated Ca^2+^ channels (VGCCs). (E) UMAP embedding based on the Ca^2+^ signaling transcriptome of RGCs, illustrating regional separation into midbrain, dorsal telencephalon (*EMX1+*), and ventral telencephalon (*EMX1-*). (F and G) Violin plots (F) and scatterplots (G) showing differentially expressed Ca^2+^-genes across the three RGC regions. (H) UMAP embedding based on the Ca^2+^ signaling transcriptome of *EMX1+* RGCs, colored by subclusters (top), with RNA velocity vectors overlaid (bottom). (I) UMAP embeddings showing neuronal and glioblast fate probabilities across *EMX1*+ RGCs. (J) Violin plots show the differential enrichment of Ca^2+^-genes across *EMX1*+ RGC subclusters. (K) Pie charts show the 10 functional classes, derived by grouping related Ca^2+^ functional groups, of the top 50 Ca^2+^-genes per subcluster.

The lineage-specific organization of the Ca^2+^ signaling transcriptome during postnatal neurogenesis inevitably raises the question of whether these genes exhibit similar trends during embryonic development. We therefore analyzed subsets of a scRNA-seq brain dataset generated from human embryos at 8 to 10 post-conception weeks (PCWs).^31^ We re-annotated the cells, guided by Braun, Danan-Gotthold et al.^32^ (Figure S8), and employed a similar Ca^2+^-factor-based workflow as for the adult mouse nervous system, with the addition of removing cell-cycle-driven Ca^2+^-factors (Figures S9A–S9C; STAR Methods). We found that the cell types were predominantly organized along both a vertical glioblast-to-neuronal axis and a horizontal excitatory-to-inhibitory axis, with an apparent regional separation within both axes (Figure 4A). The Ca^2+^-states were largely congruent with the cell types, capturing most of the heterogeneity in the dataset (Figure S9D). The distribution of cell-factor scores indicated that most neuronal Ca^2+^-factors were cell-type-specific, with, for example, *Ca*^*2+*^*-factor-3*, *-7*, *-9*, and *-14* marking the CB inhibitory neurons, neocortical pyramidal neurons, CB granule neurons, and diencephalic inhibitory neurons, respectively (Figure 4B). Similarly, within the early progenitors, the Ca^2+^-factors were enriched in a region-dependent manner (Figure 4C). The cell-gene scores for the VGCCs, CAMKs, CRAC channels, and ATPases showed that individual members were differentially enriched across the cell states (Figure 4D). Within the VGCCs, for example, *CACNA1H* was specific for the entire *EMX1+* lineage, and *EMX1+* neurons were further enriched for *CACNA1E* and *CACNA1A*. In contrast, *CACNA1C*, *CACNA1D*, and *CACNA1G* were more specific in neuroblasts and neurons of the mid- and hindbrain regions.

Given the regional specificity of the Ca^2+^-factors within the early progenitor populations and the implication of Ca^2+^ signaling in driving RGC function,^8^^,^^33^ we next sought to investigate regional differences in the expression of Ca^2+^-genes in RGCs. Although we annotated cell populations as either “RGCs” or “glioblasts,” there were recurring signs of cell variability within both populations (Figure S10A). Accordingly, we identified RGCs by their expressions of *HES1, SOX2,* and *NES,* and the absence of *BCAN* and *NHLH1* (Figures S10B–S10D, Methods). The RGCs were predominantly from the *EMX1+* dorsal telencephalon, which eventually develops into the neocortex, the *EMX1-*ventral telencephalon, and the midbrain (Figure 4E). Interestingly, distinct sets of Ca^2+^-genes were upregulated in each developmental compartment (Figures 4F and 4G), with, for example, *CREB5* marking the telencephalon, *ADGRV1* and *NTRK3* specifically marking the ventral and dorsal telencephalon, respectively, and *CXCR4* marking the midbrain.

Because RGCs in the neocortical ventricular zone are recognized for their spontaneous Ca^2+^ oscillations with varying frequencies and amplitudes during embryonic development,^33^ we hypothesized that *EMX1*+ RGCs exhibit transcriptional variations in their Ca^2+^-genes. By reiterating our clustering strategy on the RGCs (Figure S10E, Methods), we identified five distinct subclusters within RGCs (Figure 4H). Each of the subclusters was enriched for different Ca^2+^-factors (Figure S10F), and RNA velocity vectors indicated that the RGCs were organized along a maturity gradient from *Subcluster-0* toward *Subcluster-1* (Figure 4H). Moreover, computing the transition probabilities toward glioblasts or neurons based on the Ca^2+^-genes (Figure S10G) indicated that *Subcluster-0* and *Subcluster-1* exhibited the highest differentiation potential toward neurons and glioblasts, respectively (Figure 4I), consistent with a neurogenic-to-gliogenic shift. Notably, our analysis identified multiple subcluster-specific Ca^2+^-genes, such as *RASGRP1, KCNIP1, CAPN9, ESYT1*, and *PDE1A* (Figure 4J).

Next, we scored the Ca^2+^-genes for each subcluster and plotted the distribution of the top 50 genes, organized by their functional classes (Table S3). Across all subclusters, “channels,” “sensors,” and “enzymes & processes” constituted the dominant functional classes (Figure 4K). Notably, however, clear differences emerged between subclusters. For example, comparing *Subcluster-0* and *Subcluster-1*, *Subcluster-0* exhibited a higher proportion of “channels” (24% versus 12%), potentially reflecting its more neurogenic character.

Beyond the Ca^2+^-genes, differential expression analysis between *Subcluster-0* and *Subcluster-1* revealed that genes expressed in late RGCs, such as *HOPX* and *MOXD1*, were upregulated in *Subcluster-1*, while genes expressed in early RGCs, including *NEUROG2* and *DLL1*, were upregulated in *Subcluster-0* (Figure S10H). This indicates that the two subclusters consist of late and early RGCs, respectively, consistent with the divergence of differentiation trajectories predicted by the Ca^2+^-genes. Furthermore, this underscores the biological relevance of transcriptional variations in Ca^2+^-genes among RGCs and provides an additional example of how differences in the Ca^2+^-gene expression can offer insight into underlying differences in lineage decisions and cell fate specification.

### Delineation of Ca^2+^ dynamics during human neocortical development

**Figure 5:**
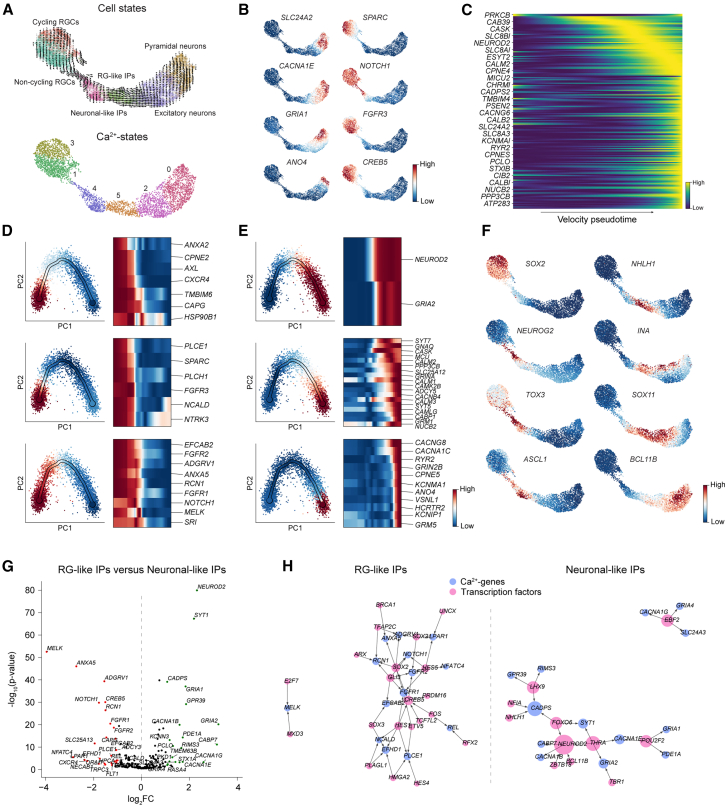
Delineation of Ca^2+^ dynamics during human neocortical development (A) UMAP embedding based on the Ca^2+^ signaling transcriptome showing the developmental trajectory of the entire *EMX1*+ lineage from van Bruggen et al.,^31^ colored by cell state with RNA velocity vectors overlaid (top) and by Ca^2+^-state (bottom). (B) UMAP feature plots show representative Ca^2+^-genes associated with the initial and terminal states. (C) Heatmap shows large-scale temporal changes in the Ca^2+^ signaling transcriptome along the pseudotime trajectory. (D and E) Examples of the 23 Ca^2+^-gene programs identified along neocortical development using scFates, highlighting early (D) and late (E) programs. (F) UMAP feature plots show the expression of key regulatory TFs associated with proliferation (left column) and differentiation (right column). (G) Volcano plot of Ca^2+^-genes upregulated in differentiating neuronal-like IPs (green) and proliferative RG-like IPs (red). (H) TF-Ca^2+^-gene networks show differentially expressed TFs and their predicted target genes in RG-like and neuronal-like IPs.

Despite numerous studies highlighting the role of Ca^2+^ signaling in the regulation of neocortical development, the specific Ca^2+^-genes involved and how these genes are remodeled along neurogenesis to adapt functionally remain unresolved. To address these questions, we analyzed the Ca^2+^ dynamics in the *EMX1*+ lineage. We found that the Ca^2+^-states closely aligned with annotated cell types while also revealing substantial state-level heterogeneity. Moreover, the Ca^2+^-genes accurately reconstructed the developmental trajectory from RGCs to pyramidal neurons via intermediate progenitor (IP) populations (Figure 5A), with RGCs representing the initial states and pyramidal neurons the terminal states (Figure S11A). Notably, this could not be attributed to differences in the number of expressed Ca^2+^-genes or the total Ca^2+^-gene-counts across cell states (Figure S11B). Instead, our results suggested that the trajectory was driven by the up- and down-regulation of different Ca^2+^-genes over time (Figures 5B and 5C). To test this hypothesis, we clustered the Ca^2+^-genes that were significantly associated with the neocortical trajectory based on their pseudotemporal kinetics (methods). We found a total of 106 Ca^2+^-genes that grouped into 23 gene regulatory programs along the developmental trajectory (Figures 5D, 5E, and S11C). Most gene programs consisted of genes that were either expressed in RGCs and thereafter downregulated, or genes that were upregulated during development, peaking at different stages. Notably, some of the early gene programs remained temporally active in the earlier IPs, while some of the neuronal gene programs had already been switched on in the later IPs. Furthermore, certain gene programs were either transiently upregulated or downregulated, and a few genes, including *CREB5, KCNIP4*, and *CACNA2D1*, each formed their own group.

Examining the Ca^2+^-driven differences between the IPs more closely, we found that the boundaries between the Ca^2+^-states aligned well with those from the whole genome and matched the switching of TFs that occurs as IPs transition from a proliferative, self-renewal state expressing *SOX2, NEUROG2*, *TOX3*, and *ASCL1,* to a differentiating state expressing *NHLH1, INA, SOX11,* and *BCL11B* (Figure 5F), as reported elsewhere.^32^^,^^34^ Moreover, differential expression analysis revealed that earlier IPs expressed RG-like Ca^2+^-genes while the latter IPs expressed neuronal-like Ca^2+^-genes (Figure 5G). Many of the Ca^2+^-genes appeared to be regulated by *SOX2* and *NHLH1*, among others (Figure 5H), implicating a role of Ca^2+^ signaling in regulating the phenotypic switch from proliferation toward differentiation.

### Conservation of Ca^2+^ dynamics during human neocortical development

**Figure 6:**
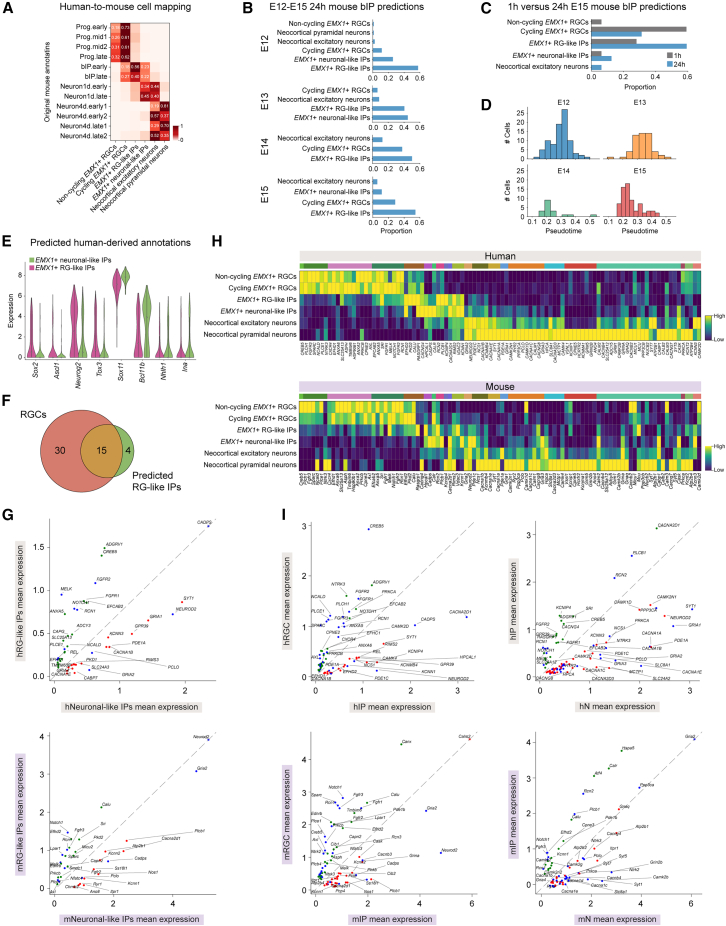
Human-defined Ca^2+^ dynamics are conserved during mouse neocortical development (A) Confusion matrix shows the distribution of predicted human-defined cell states across mouse neocortical populations from Telley et al.^36^ using *scArches*. Each row is normalized to sum to 1; only values > 0.1 are shown. (B and C) Bar plots show the predicted cell state distributions of 24-h-old mouse IPs, stratified by birth date (E12–E15) (B) and 1-h-old versus 24-h-old E15 mouse IPs (C). (D) Histograms of pseudotime values for mouse IPs, stratified by birth date, highlighting a progressive leftward shift with later birth dates. (E) Violin plots of mouse IPs grouped by their predicted human-defined cell states, showing the expression of non-Ca^2+^-gene TFs consistent with the predicted identities. (F) Venn diagram showing the overlap of differentially expressed genes between mouse RGCs and predicted RG-like mouse IPs. (G) Scatterplots compare RG-like IPs and neuronal-like IPs in human (top) and mouse (bottom). Each point represents the mean gene expression within each population. Blue dots indicate conserved Ca^2+^-genes, green dots indicate genes significantly upregulated in the developmentally more immature population, and red dots indicate genes significantly downregulated. Only differentially expressed Ca^2+^-genes are shown. (H) Matrix plot shows the expression of neocortical Ca^2+^-gene programs in human (top) and mouse (bottom) datasets, grouped by cell state. Each row represents the mean expression per cell population, and each column represents a gene. Genes are color-coded by gene program. (I) As in (G), comparing RGCs versus IPs (left) and IPs versus neurons (N; right), shown for human (top) and mouse (bottom) datasets.

To assess the evolutionary conservation of Ca^2+^-gene dynamics during human neocortical development, we transferred human-defined cell types^35^ to a mouse neocortical dataset from Telley et al.^36^ using our Ca^2+^-genes. This dataset spans embryonic day 12 (E12) to postnatal day 0 (P0) (Figures 6A and S12A; Table S7). A substantial fraction (87–94%) of mouse apical progenitors was classified as RGCs. Likewise, the majority (87–100%) of 4-day-old neurons mapped to one of the two neuronal categories, indicating high mapping accuracy. Notably, we observed considerable overlap between 1-day-old neurons and IP populations. Approximately half of the 1-day-old neurons (46–56%) were predicted to be IPs, predominantly neuronal-like. Despite differing from the original annotations, these cells retained higher expression of *Eomes* (Figure S12B), consistent with the Ca^2+^-driven mapping.

Although mouse IPs were originally described as two age-defined populations, both groups exhibited similar overall distributions of predicted cell states (Figure 6A). However, stratification by birth date revealed that IPs born at E12-E13 were generally more mature than those born at E14-E15 (Figures 6B and S12C). Furthermore, comparisons of 1-h-old and 24-h-old IPs within the E15 cohort showed that a larger proportion of the younger cells were classified as RGCs (65% versus 29%), whereas the older cells were predominantly classified as RG-like IPs (54% versus 28%) (Figure 6C). Re-analysis of mouse IPs revealed their organization along a pseudotime axis that inversely correlated with birth date (Figure 6D). Predicted neuronal-like IPs exhibited higher expression of neuronal TFs such as *Neurod2*, *Nhlh1*, and *Bcl11b*, whereas predicted RG-like IPs showed increased expression of *Sox2, Ascl1*, and *Hes1* (Figures 6E and S12D). Consistent with their RG-like state, predicted mouse RG-like IPs displayed the upregulation of 19 Ca^2+^-genes, 15 of which were also upregulated in mouse RGCs (Figure 6F), including *Sparc, Notch1*, *Fgfr1, Fgfr2, Lpar1*, and *Rcn1* (Figure 6H). Together, these findings further support the accuracy of the Ca^2+^-driven cross-species mapping.

To directly assess the conservation of the Ca^2+^-genes, we compared the expression of the identified neocortical developmental Ca^2+^-gene programs in human and mouse. We found strong consensus across the species, suggesting that these Ca^2+^-gene programs were largely conserved, although a few genes showed contrasting expression patterns (Figures 6H and S12E). Furthermore, in our analysis of differentially expressed Ca^2+^-genes across each sequential developmental step, we consistently observed that the most enriched genes, including *CREB5* and *NCALD* for RGCs, *CACNA2D1* and *CADPS* for IPs, and *CACNA1A* and *PPP3CA* for neurons, were conserved (Figure 6I), although the degree of conservation varied among cell types (Figure S12F). Collectively, these findings highlight that Ca^2+^-genes not only capture nuanced variations in cell states but also demonstrate a significant degree of evolutionary conservation of Ca^2+^ signaling in neocortical development, further emphasizing the role of specific Ca^2+^ signaling pathways in deterministically controlling cell states.

## Discussion

Here, we have comprehensively examined the Ca^2+^ signaling transcriptome in the entire adult mouse nervous system and early human brain development. Despite Ca^2+^ being a ubiquitous intracellular signaling mediator involved in regulating virtually all neuronal functions, our results reveal that the Ca^2+^ signaling transcriptome governing the Ca^2+^ signaling protein toolkit is heterogeneously and dynamically organized across the brain. Distinct neuronal types express specific combinations of the Ca^2+^ signaling protein toolkit, likely reflecting differences in firing properties, cell fate decisions, and stages of functional maturation. This was exemplified by the neocortical layer II-IV neurons segregating into distinct Ca^2+^-states associated with differences in experience-dependent remodeling, as well as Ca^2+^-states capturing neurogenic-to-gliogenic shifts in cortical RGCs and the separation of IPs attributable to the phenotypic switch from proliferation to differentiation. These findings suggest that Ca^2+^ signaling diversity is not solely determined by dynamic intracellular Ca^2+^ fluctuations but is partly encoded at the transcriptional level through cell-state-specific combinations of Ca^2+^-regulatory genes. Such transcriptional “Ca^2+^ signaling programs” may therefore represent a previously underappreciated layer of regulation linking developmental lineage, neuronal identity, and signaling capacity. Although our study is the first to specifically study the diversity in the Ca^2+^ signaling transcriptome across the brain, a survey of recent scRNA-sequencing studies focused on profiling neuronal cell types across different time points and regions revealed a recurrence of Ca^2+^-genes used as population-defining transcriptional markers, such as *Cacna2d1* in mouse interneurons,^37^ *CALB1* in the human midbrain,^38^ *PVALB* specifically in human midbrain glutamatergic neurons,^11^ and *CACNB2* and *CACNG3* for OPCs in early human development.^32^ These studies are consistent with our findings and support the hypothesis that the diversity of the Ca^2+^ signaling transcriptome has previously been underestimated.

Naturally, the heterogeneity of Ca^2+^ signaling in neurons raises the question of whether it is a function of development, whether there may exist population-specific environmental factors that influence the transcriptional Ca^2+^ state of neurons, or whether they reflect underlying neural circuitry. The ability of Ca^2+^-genes to reconstruct and differentiate between developmental trajectories using lineage-specific combinatorial patterns of Ca^2+^-gene programs illustrates that the divergence in Ca^2+^-states already begins to be specified during the progenitor stages, and thus that neurons are likely inherently primed to express specific subsets of Ca^2+^-genes. This is further supported by the large-scale heterogeneity observable in early human brain development, where cells ranging from RGCs to neurons express region- and lineage-specific Ca^2+^-genes. With previous reports implicating Ca^2+^ signaling in regulating neurogenesis across many regions,^9^^,^^39^ this transcriptional diversity suggests that the Ca^2+^-driven mechanisms are likely lineage-specific. Our results indicate that they may be directly activated by key regulatory TFs. Simultaneously, the clear regional patterning in Ca^2+^-genes observable in progenitors as well as neurons suggests that environmental factors influence the transcriptional output and may potentially reflect the interactions between Ca^2+^ signaling, morphogens, and TFs.

Within neocortical development, many Ca^2+^-genes are highly dynamic and strictly regulated, thereby containing a tremendous amount of information, as seen by the accurate reconstruction of the lineage, and capturing key state-specific differences within RGCs and IPs. Our findings implicate numerous previously unexplored genes, including *CREB5*, *NCALD, KCNIP4*, and *CACNA2D1*, which appear to be evolutionarily conserved. Together, our findings align with previous studies showing the critical role of Ca^2+^ signaling in regulating various aspects of neurogenesis, including cell proliferation, differentiation, and migration.^33^^,^^39^ However, our study adds a new layer of understanding by elucidating the lineage-specific dynamics of Ca^2+^ signaling, with important implications for neocortical development, and identifies new regulators that warrant future investigation in functional studies of neurogenesis and neuronal maturation.

In conclusion, our study reveals substantial diversity in the Ca^2+^ signaling transcriptome across the human and mouse brain and shows that differential Ca^2+^ signal output is not driven solely by the spatiotemporal dynamics of Ca^2+^ itself, but also reflects cell state-specific functional priming that shapes and constrains potential signaling outputs. This is evidenced by the ability of Ca^2+^-genes to capture fine-grained differences despite comprising only hundreds of genes. Accordingly, Ca^2+^ signaling may be more accurately viewed as a dynamic signaling system that is tailored to specific neuronal identities while also being modulated by cell state-dependent functional priming within neuronal cell types.

### Limitations of the study

There are several limitations to our study, particularly in linking expression patterns to functional outcomes. Current methodologies allow the measurement of Ca^2+^ signaling in only a limited number of cells within thin brain sections. Addressing this will require increased throughputs to enable three-dimensional monitoring of Ca^2+^ signaling across large neuronal populations. Moreover, we cannot determine whether the observed up- or downregulation of Ca^2+^ signaling genes at key developmental stages actively drives developmental progression or merely reflects it. Likewise, the regulatory networks that position Ca^2+^ signaling genes as downstream targets remain to be experimentally validated. In addition, more detailed analyses of specific Ca^2+^ signaling pathways within individual cell types may uncover genes critical for distinct cellular functions. A further limitation concerns sex-based generalizability: Because individual cell-level sex annotations were not available in the source datasets, sex-disaggregated analyses could not be performed, and whether the Ca^2+^ signaling transcriptome differs between sexes therefore remains an open question. Nevertheless, the primary aim of this study was to provide a comprehensive, brain-wide analysis of the Ca^2+^ signaling transcriptome to define the molecular logic underlying signaling specificity, an objective that, to our knowledge, has not previously been achieved.

## Resource availability

### Lead contact

Requests for further information should be directed to the lead contact, Professor Per Uhlén (E-mail: per.uhlen@ki.se).

### Materials availability

This study did not generate new materials.

### Data and code availability

• This paper analyzes existing, publicly available data. Accession numbers are listed in the key resources table.
• This paper does not report original code. Any scripts or notebooks used to generate figures are available from the lead contact upon request.
• Any other information required to reanalyze the data reported in this paper is available from the lead contact upon request.

## Acknowledgments

We would like to thank Dr. David van Bruggen for valuable discussions throughout this study. A special thanks to Dr. Sten Linnarsson, Dr. Gonçalo Castelo-Branco, and Dr. Peter Lönneberg for providing access to data and their high-performance cluster and Uhlén lab members for critical feedback. This work was funded by: 10.13039/501100004359Swedish Research Council (2021-03108 and 2025-02414 to P.U.), 10.13039/501100004359Swedish Research Council (2022-02185 to E.S.), 10.13039/501100003792Swedish Brain Foundation (FO2020-0199 and FO2024-0057-TK-138 to P.U.), Swedish Cancer Society (22 2454 Pj and 25 4957 Pj to P.U.), and 10.13039/501100004047Karolinska Institutet CSTP doctoral program (to I.A.R. and I.D.E.).

## Author contributions

Designed the study: I.A.R., L.L., E.S., and P.U. Data curation: I.A.R. and I.D.E. Analyzed the transcriptional data: I.A.R. Wrote the paper: I.A.R. and P.U. Oversaw and conceived the study: P.U.

## Declaration of interests

The authors declare no conflict of interest.

## STAR★Methods

### Key resources table

**Table:** 

REAGENT or RESOURCE	SOURCE	IDENTIFIER
**Deposited data**		

Atlas of the entire mouse nervous system	Zeisel et al.^14^ http://mousebrain.org/adolescent/downloads.html	https://doi.org/10.1016/j.cell.2018.06.021 Accession code SRP135960
Atlas of human forebrain development	van Bruggen et al.^31^ https://cells.ucsc.edu/?ds=human-forebraindev	https://doi.org/10.1016/j.devcel.2022.04.016 Accession code S00001006136
Dataset of the developmental mouse neocortex	Telley et al.^36^	https://doi.org/10.1126/science.aav2522 Accession code GSE118953

**Software and algorithms**		

CellRank	Lange et al.^30^	https://github.com/dpeerlab/cellrank
gget	Luebbert et al.^40^	https://github.com/pachterlab/gget
harmonypy	Korsunsky et al.^41^	https://github.com/slowkow/harmonypy
MetaNeighbor	Crow et al.^18^	https://github.com/gillislab/MetaNeighbor
optuna	Akiba et al.^42^	https://github.com/optuna/optuna
pySCENIC	Aibar et al.^43^	https://github.com/aertslab/pySCENIC
scanpy	Wolf et al.^44^	https://github.com/scverse/scanpy
scArches	Lotfollahi et al.^35^	https://github.com/theislab/scarches
scFates	Faure et al.^45^	https://github.com/LouisFaure/scFates
scHPF	Levitin et al.^15^	https://github.com/simslab/schpf
scikit-learn	Pedregosa et al.^46^	https://github.com/scikit-learn/scikit-learn
scVelo	Bergen et al.^47^	https://github.com/theislab/scvelo

### Experimental model and study participant details

#### Adult mouse nervous system dataset

Data from Zeisel et al.^14^ were used to characterize the Ca^2+^ signaling transcriptome in the adult mouse nervous system. Raw unique molecular identifier (UMI) counts (spliced and unspliced) and cell metadata were obtained directly from the authors. The original dataset was generated from male and female mice aged postnatal day 12 to 8 weeks, under ethical approval from the Stockholms Norra Djurförsöksetiska nämnd, Sweden; full details on strains, husbandry, and tissue isolation are reported in the original publication. No new animal experiments were performed in the present study. Non-neuronal cells were excluded, yielding a final dataset of 73,603 neurons classified into 214 cell types. Donor sex was not annotated at the cell level in the source data; this limitation is addressed in the Discussion.

#### Developing human brain dataset

Data from van Bruggen et al.^31^ were used to investigate Ca^2+^ signaling dynamics in early human brain development. The original dataset was derived from human first-trimester forebrain tissue (8 to 11 weeks post-conception) obtained from voluntary elective abortions with written informed consent, under ethical approval from the Swedish Ethical Review Authority (ref. 2007/1477-31/3 and amendments) and the National Board of Health and Welfare; full procedural details are reported in the original publication. No new human samples were collected for the present study. Only cells generated with 10X Genomics Chromium version 3 were retained to avoid batch correction artefacts, yielding 12,990 cells from post-conception week (PCW) 8.5 to 9. Cell annotations were assigned based on comparisons with Braun, Danan-Gotthold et al.^32^ and the published literature. Donor sex was not reported in the source dataset; this limitation is addressed in the Discussion.

### Method details

#### Terminology conventions

To ensure conceptual clarity, we use the following terminology consistently throughout this study:

• Ca^2+^-genes refer to the curated set of genes encoding proteins that regulate, transduce, or execute Ca^2+^ signaling.
• Ca^2+^ signaling transcriptome denotes the collective expression of Ca^2+^-genes within a cell or cell population and is used to describe system-level signaling properties rather than individual genes; it represents the transcriptional deployment of the Ca^2+^ signaling protein toolkit.
• Ca^2+^-factors refer to latent dimensions inferred by single-cell hierarchical Poisson factorization from the expression of Ca^2+^-genes; cell-factor and gene-factor scores denote the corresponding loadings of cells or genes on these factors.
• Ca^2+^-states denote biologically interpretable clusters derived from the Ca^2+^-factors that reflect stable cellular Ca^2+^ signaling configurations.
• Subclusters refer to Ca^2+^-states identified within a restricted cell population.
• Functional groups denote curated, fine-grained categories of Ca^2+^-genes defined by shared molecular mechanisms or signaling roles (e.g., voltage-gated Ca^2+^ channels, ligand-gated Ca^2+^ channels, Ca^2+^/CaM Kinases).
• Functional classes refer to higher-order groupings obtained by aggregating related functional groups into broader categories (e.g., Channels, Sensors, Receptors).
• Gene programs refer to coordinated, temporally ordered patterns of gene regulation, typically identified along developmental trajectories, and should not be conflated with Ca^2+^-factors.
• Cell types refer to taxonomically defined cellular identities based on stable transcriptional and functional characteristics, whereas cell states refer to dynamic transcriptional configurations within a given cell type, such as developmental stage, maturation, or activity-dependent variation.

#### Selection of Ca^2+^-genes

The Gene Ontology Consortium annotations were searched using AmiGO 2 to find genes associated with Ca^2+^ signaling (referred to as the Ca^2+^-genes) using the keyword “Calcium” and restricted to the organisms “Homo sapiens” and “Mus musculus”. Annotations with the evidence code equivalent to only “automatic assertion” or “with no evidence using manual assertion” were excluded, yielding 1,515 genes. The GO compiled list was merged with the 1,670 genes found in the Calcium Gene Database (CaGeDB), our previously developed online database that maps genes related to Ca^2+^ signaling and their associated diseases,^48^ totaling ∼2,000 genes, followed by checking for the inclusion of canonical Ca^2+^-genes. The list was finally manually curated by surveying the genes against UniProt, GeneCards, and published literature to exclude genes with unclear links to Ca^2+^ signaling, yielding a final list of 604 Ca^2+^-genes. The genes were subsequently annotated and grouped into 46 functional groups (Table S3), each containing at least 5 genes.

#### Characterization of mouse Ca^2+^-states

The total UMI count matrix (i.e., sum of spliced and unspliced) across all Ca^2+^-genes was decomposed using the *scHPF* package^15^ with default settings, generating two matrices representing cell- and gene-factor scores across *K* factors. Guided by the selection criteria outlined in the *scHPF* package (https://schpf.readthedocs.io/en/latest/select-k.html), multiple iterations of the *scHPF* pipeline were run with *K* set to the maximum number of factors, where, on average, four factors captured a minimum of 70% of the cells’ scores (Figures S1A and S1B), yielding *K* = 27 for the mouse nervous system dataset. Mutual information scores were used to assess factor redundancy (Figure S1C). Unless otherwise stated, all downstream analysis and visualization was done through *scanpy*.^44^ Cells were clustered on the cell-factor scores by constructing a *k*-nearest neighbors (KNN) graph on the Jensen-Shannon distance (JSD) between every cell (*k* = 15) and neighbors were pruned by defining an information radius around every cell equal to the 85^th^ percentile of distances between neighboring cells in the KNN graph. The Leiden algorithm was used to generate clusters with the resolution set at 1.5. Clusters with <20 cells were deemed as outliers and removed, yielding 33 initial clusters. To ensure that the clusters used in the downstream analysis were robust and represented stable, interpretable transcriptomic states, the clusters were further refined by 1) removing cells assigned to a cluster if <10% of the cells or <15 cells belonging to that cell type were found there, and 2) merging clusters with <250 cells into a neighboring cluster based on a dendrogram constructed on high-dimensional UMAP coordinates (Figure S1D). This yielded a final set of 63,507 cells, clustered into 28 different Ca^2+^-states. For the UMAP, the kNN-graph was re-computed using the JSD with *k* = 50 without radius pruning.

#### Characterization of human Ca^2+^-states

To characterize the Ca^2+^-states in human, *scHPF* was run with the same criteria as for the adult mouse data, yielding 31 Ca^2+^-factors. To remove cell-cycle-related Ca^2+^-factors, Pearson correlations were calculated between cell-factor scores and the cell cycle markers *TOP2A, MKI67,* and *BIRC5*. *Ca*^*2+*^*-factor-11* and *Ca*^*2+*^*-factor-16* were thus excluded from further analysis due to their high correlation coefficients, yielding 29 final Ca^2+^-factors. A clustering strategy similar to that used for the adult mouse was subsequently applied (neighbors = 15, information radius defined by the 85^th^ percentile), with the Leiden resolution set to 2.5 and clusters containing fewer than 20 cells removed.

#### *scHPF* analysis and performance comparisons

A subset of 20% of genes from the dataset was used for assessment of the computational efficiency. The genes were pre-filtered such that only genes expressed in at least 10 cells were included, and all Ca^2+^-genes were excluded to avoid overlap in the gene sets. A sampling strategy was devised to randomly sample genes while simultaneously attempting to control for differences in expression levels and spread between Ca^2+^-genes and random genes. For each Ca^2+^-gene, the mean expression was calculated, and an expression interval equal to a deviation of 10% was allowed. From all of the expressed genes, genes with a mean expression outside of this interval were filtered out, followed by randomly sampling a gene from the remaining ones. The deviation was added to ensure that each iteration of *scHPF* was running on a different set of random genes. To assess the performance of the iterations, the cells were labeled as their respective Ca^2+^-states and projected on UMAPs. The local inverse Simpson’s Index score (LISI)^41^ was computed for each UMAP embedding through the package *harmonypy*^41^ and was used to compare the performance of each iteration. Briefly, for each cell, LISI measures the diversity in the local neighborhoods of cells, where a score of 1 implies a homogenous neighborhood and higher scores imply a mixture of multiple neighborhoods.

#### Random forest classifier construction

The Python packages *scikit-learn*^46^ and *optuna*^42^ were used to build our random forest classifiers. Both the Ca^2+^ and random gene counts were first normalized and log-transformed. The classifiers were trained on 80% of the data, and performance was evaluated by classifying the remaining 20% held out. For each classifier, *optuna* was used for optimization, with ‘n_trials’ set to 100, and StratifiedShuffleSplit was used for cross-validation by generating multiple randomized folds (n_splits = 5). For both the random genes and the Ca^2+^-genes, separate classifiers were built to predict Ca^2+^-states and cell types. As described above, the random genes were matched to the Ca^2+^-genes for the classifiers by selecting genes with the lowest differences between the means compared to Ca^2+^-genes.

#### Computation of cell-gene scores

To transform the cell-factor and gene-factor matrices back into a cell-gene matrix, we computed the dot product of the cell-factor scores and the gene-factor scores, yielding the Poisson rate matrix that served as a cell-gene count matrix. For visual purposes, the resulting counts were z-normalized. For each Ca^2+^-state, the top Ca^2+^-genes were identified by ranking genes according to their mean expression across all cells within that Ca^2+^-state. Unless otherwise stated, the top 50 genes were used.

#### Gene regulatory network analysis

For the computation of enriched gene regulatory networks, the single-cell regulatory network inference and clustering (SCENIC) pipeline was run through the Python package *pySCENIC*.^43^ Genes expressed in <3 cells were first removed. The parameter “auc_threshold” was set at 0.01, and “nes_threshold” was set at 2.0, with the rest kept at default. For the developmental human and mouse data, the Ca^2+^-genes were merged with the TF list provided in the package to capture potential Ca^2+^-genes with promoter binding activity. For the adult mouse data, the built-in regulon specificity score (RSS) function was used to find enriched regulons. For each of the two Ca^2+^-states, the top 20 TFs were selected. For each TF, the top 30 targets were selected, and only the differentially expressed were retained in the final comparison. The Spearman correlation was calculated across the entire dataset and included all of the predicted TFs. The TF-target gene networks were constructed using the networkx package.

#### Lineage inference in mouse adult brain

For the lineage inference analysis across the developmental subset of the mouse brain data, the data were first subsetted to include the developmental Ca^2+^-states. Next, *scHPF* was re-run with *K* instead tuned such that 3 Ca^2+^-factors on average captured 70% of the cells’ scores, yielding 18 Ca^2+^-factors. The KNN graph using the JSD was set at *k* = 100 and the Leiden resolution at 0.6. Clusters belonging to neither lineage were then removed. The lineage trajectory inferences were computed using the *CellRank*^30^ and *scVelo*^47^ packages. Standardized gene filtering and count matrix normalization were done (min_cells = 3) prior to the selection of Ca^2+^-genes, yielding 15,199 cells expressing 469 Ca^2+^-genes. Moments were calculated using ‘scvelo.pp.moments’ with n_pcs = 15 and n_neighbors = 30, and the velocity vectors were calculated with the mode set to ‘stochastic’. CellRank was then run with the default setting using a combined kernel weighting the “VelocityKernel” and “ConnectivityKernel” 80% to 20%. The lineage-associated TFs and Ca^2+^-genes were subsequently computed through the built-in function compute_lineage_drivers (method = ‘perm_test’).

#### Lineage inference in human *EMX1+* cells

First, the *EMX1+* RGCs, neuronal intermediates, and neurons were selected. Next, the cells were clustered based on Ca^2+^-genes, with the following parameters: number of neighbors = 15, distance = correlation, and Leiden resolution = 0.6. Following this, the lineage inference was done using the same settings for the mouse data outlined above. To relate RGCs to a potential glioblast or neuronal fate, the inference analysis pipeline was re-run with the inclusion of *EMX1+* glioblasts and manually setting the number of macrostates to 2 by using “g.compute_macrostates (n_states = 2)” prior to computing terminal states, ensuring that the absorption probabilities towards glioblasts would be calculated. The absorption probabilities towards neurons and glioblasts for RGCs were then *z-*score normalized and correlated with the clusters.

#### Pseudotemporal and bifurcation analysis

The pseudotemporal analysis along the neocortical *EMX1+* lineage and the bifurcation analysis of the divergence towards the DG- and OL-lineages, respectively, were done using the *scFates* package.^45^ To ensure that the significant Ca^2+^ features would not be biased, the entire pipeline was first run using all genes. For the pseudotemporal trajectory analysis on the human data, the pipeline was run using the default settings. All non-Ca^2+^-genes were removed after running the “scfates.tl.test_association” function. The parameter “A_cut” was set to the 90^th^ percentile, resulting in a total of 106 significant Ca^2+^-genes that were subsequently fitted. To find gene programs, genes were clustered based on their fitted kinetics. First, the expression was normalized such that they summed to 1. Next, the Ca^2+^-genes were hierarchically clustered using complete linkage on the cosine distances, generating 23 gene clusters that were subsequently plotted using the *scFates* plotting interface. For the bifurcation analysis, a principal tree was generated using the “ppt” method on the UMAP-embedding. Next, the branch point was extracted. “A_cut” was set to 0.4 and the genes were calculated using “scfates.tl.test_fork” and “scfates.tl.branch_specific” with effect = 0.1 resulting in a total of 28 Ca^2+^-genes (18 DG; 10 OL).

#### Cross-species neocortex integration

The Python package *scArches*^35^ with the built-in model *scPoli*^49^ was used to integrate the two datasets, using the human data as the reference and the mouse data as the query. It stands to reason that if the Ca^2+^ signaling transcriptome was generally conserved between human and mouse, mapping the mouse cells using the expression from human cells should be possible, assuming the existence of comparable cell populations across both datasets. Accordingly, all non-Ca^2+^-genes were removed, and only the shared Ca^2+^-genes were retained prior to the mapping. The integration was run using recommended settings based on the documentation tutorial, only changing n_epochs (=30) and pretraining_epochs (=24) within the function “scPoli.train”. The performance was evaluated by comparing the predicted human annotations to the original mouse annotations.

#### Analysis of human radial glial cells

To define radial glial cells (RGCs), we used the *scanpy* package function “scanpy.tl.score_genes” to calculate a combined score based on *HES1, NES,* and *SOX2*, as well as a separate score based on *BCAN* and *NHLH1*, across all cells. The scores were then binarized with a cutoff = 1. RGCs were then defined as cells with a combined *HES1, NES,* and *SOX2* score of 1, and the rest were assigned a score of 0, totaling 1501 RGCs. Differential expression analysis was done by grouping the RGCs by developmental compartment (dorsal/ventral telencephalon and midbrain). The log foldchange threshold was set at 1. For the analysis of the heterogeneity within *EMX1+* RGCs, *scHPF* was run with the Ca^2+^-factors set at 50, and 3 cycling Ca^2+^-factors were removed. Clustering was done using the JSD distance (neighbors = 25, Leiden resolution = 0.7), yielding five subclusters. Differential expression analysis between *Subcluster-0* and *Subcluster-1* was done with the log foldchange threshold set at 0.5 and used as the basis for deducing early/late RGCs. To ensure accuracy, the velocity vectors were calculated using all genes.

#### Ca^2+^-state versus cell type discrimination

To determine the relationship between *Ca*^*2+*^*-state-1* and *Ca*^*2+*^*-state-20* as well as their corresponding cell types (*TEGLU7, TEGLU8, TEGLU12*), conventional pre-processing was done through *scanpy*, including filtering lowly expressed genes, normalizing the counts and computing the most variable genes prior to running a principal component analysis (PCA). The linear discriminant analysis (LDA) was then calculated using the PCA cell loadings, with an incremental increase in the number of PCs.

### Quantification and statistical analysis

#### Enrichment of Ca^2+^ functional groups

The relative importance of Ca^2+^ functional groups in distinguishing Ca^2+^-states was assessed using *MetaNeighbor*,^18^ following the approach described by Morarach et al.^50^ Briefly, *MetaNeighbor* quantifies the ability of each functional group to discriminate between Ca^2+^-states. Functional groups containing at least five genes were included, yielding 46 individual groups (Table S3). These were further organized into 10 broad Ca^2+^ functional classes by grouping related functional groups (Table S3). Performance was quantified as the mean area under the receiver operating characteristic curve (AUROC). For visualization, only functional groups with an average AUROC greater than 0.55 were plotted (Figure 1G).

#### Enrichment of synapse remodeling genes

The comparison of synapse remodeling-related genes across the cell types was computed using a list of genes adapted from Südhof.^19^ Differential expression analysis was done with the log foldchange threshold set at 0.5.

#### Gene set enrichment analysis

The *gget* package^40^ was used to perform the gene set enrichment analyses with databases Reactome_2022 and KEGG_2021_Mouse.

## Supplemental information

Document S1. Figures S1–S12

Table S1. Assignment of cells annotated in Zeisel et al, ^14^ to their corresponding Ca^2+^-states

Table S2. Corrected cell-gene scores derived from cell-factor and gene-factor matrices

Table S3. Ca^2+^-genes grouped by canonical functional categories

Table S4. Differentially expressed Ca^2+^-genes between Ca^2+^-states

Table S5. Gene ontology enrichment analysis of differentially expressed Ca^2+^ signaling and synapse-related genes

Table S6. Differentially expressed synapse-related genes between Ca^2+^-states

Table S7. Transfer of human-defined cell type labels to the mouse neocortical dataset
