# Supplementary material for: Mapping the transcriptional diversity of calcium signaling in the mouse and human brain
Source: iScience. 2026 May 28;29(6):116055. doi: 10.1016/j.isci.2026.116055 (PMC13233772; doi:10.1016/j.isci.2026.116055)
Supplement: Document S1. Figures S1–S12 [file mmc1.pdf]

iScience, Volume *29*

Supplemental information

Mapping the transcriptional diversity of calcium signaling in the mouse and human brain

Ibrahim Al Rayyes, Lauri Louhivuori, Ivar Dehnisch Ellström, Erik Smedler, and Per Uhlén

Supplementary figures

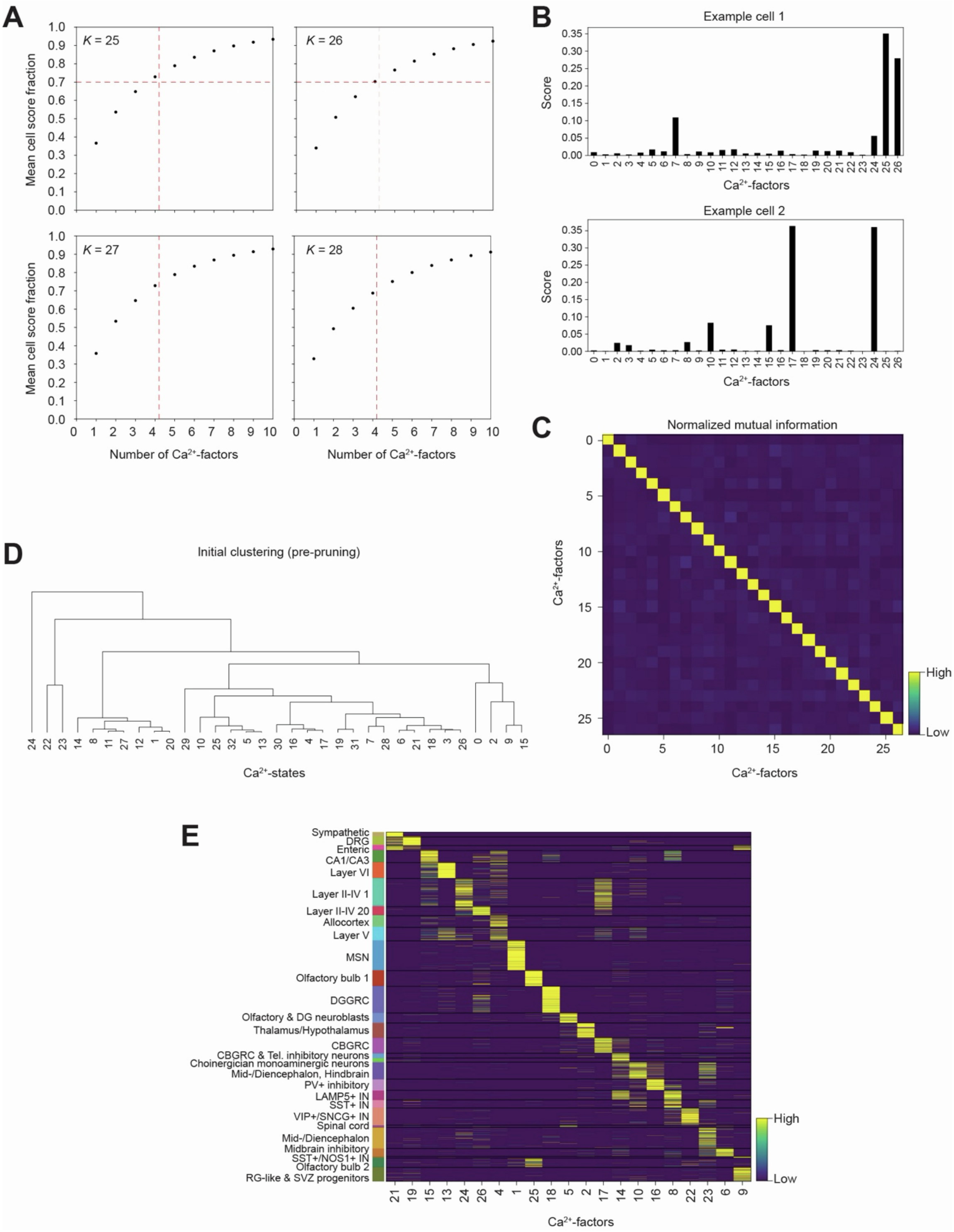

Figure S1. Overview of the HPF-based clustering strategy. (**A**) Mean cell score fraction plotted against the number of Ca^{2+}-factors. In the Zeisel *et al*. [14] dataset, *scHPF* was iteratively run with *K* manually tuned to the largest value at which four Ca^{2+}-factors captured an average of 70% of the cell scores. (**B**) Examples of cell-factor scores for two randomly sampled cells, illustrating the differential enrichment of Ca^{2+}-factors across the atlas. (**C**) Heatmap of the normalized mutual information scores between the Ca^{2+}-factors. (**D**) Dendrogram of the initial 33 Ca^{2+} clusters, pre-pruning and merging the small clusters according to the hierarchical similarities. (**E**) Heatmap showing the differential enrichment of Ca^{2+}-factors across the 28 final Ca^{2+}-states.

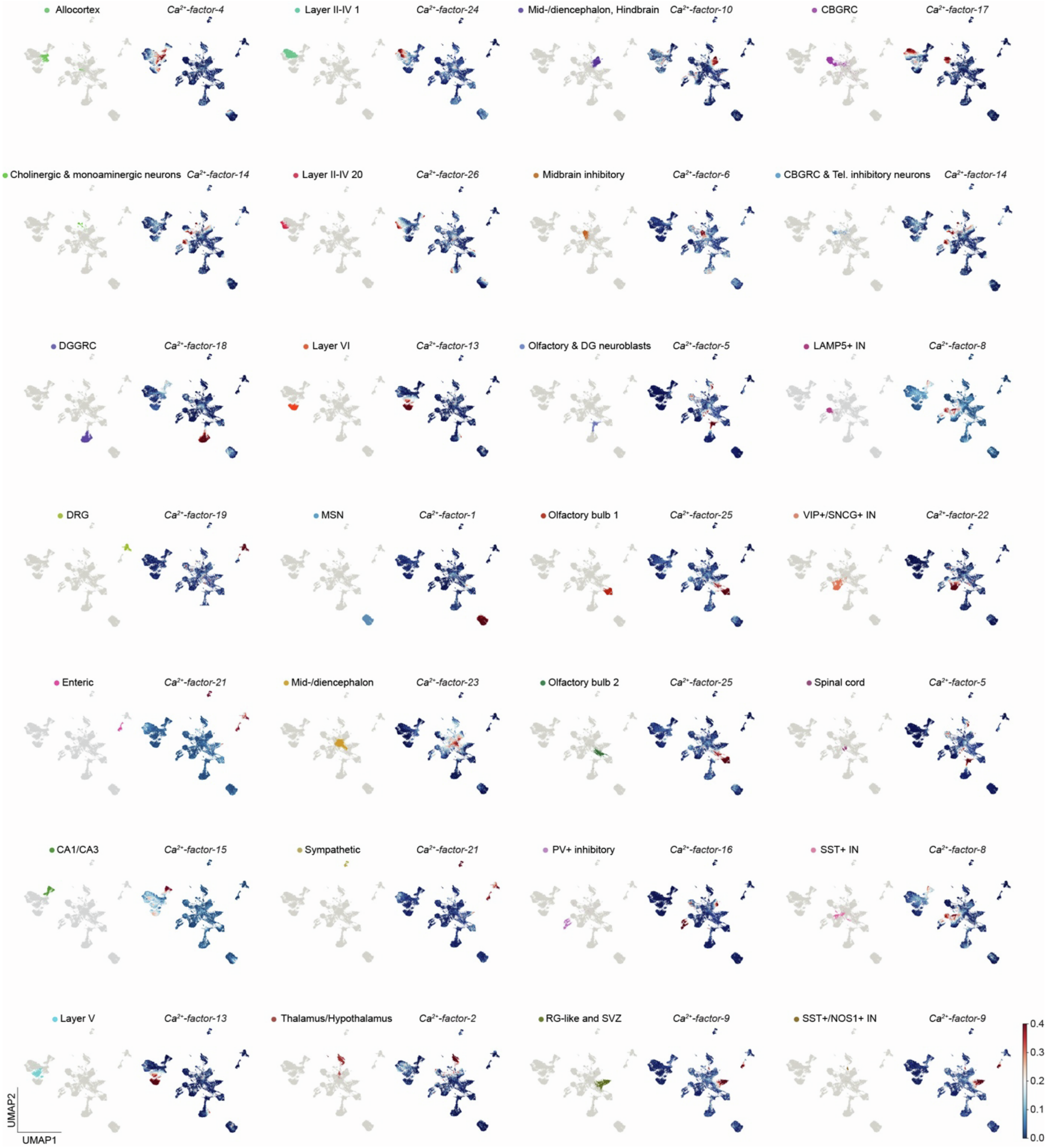

Figure S2. Illustrations of the 28 Ca^{2+}-states and their associated Ca^{2+}-factors. UMAP feature plots of each individual Ca^{2+}-state (left) and their highest associated Ca^{2+}-factor (right).

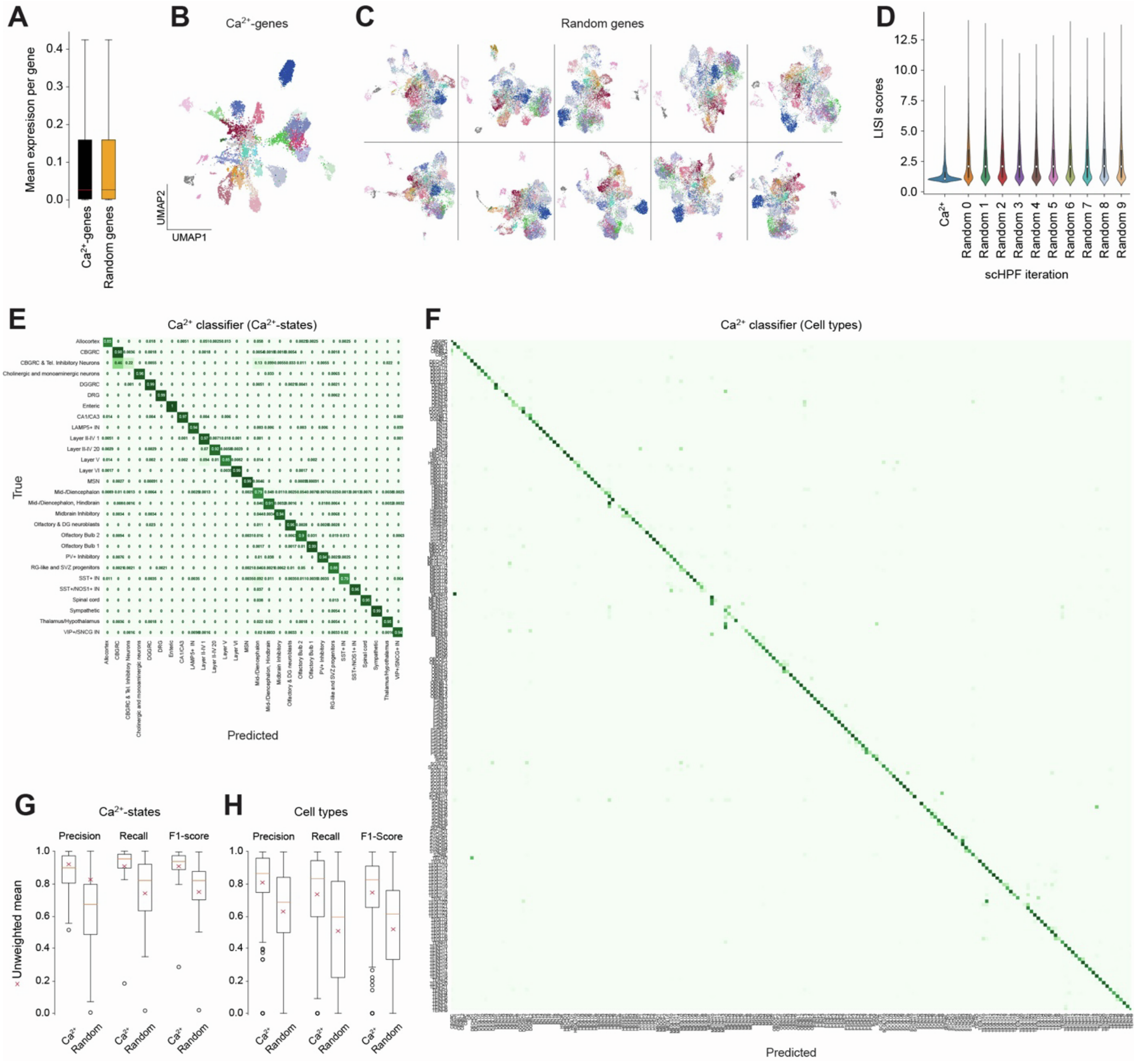

Figure S3. Comparisons between Ca^{2+}-genes and random genes. (**A**) Boxplots illustrating the expression level distributions of Ca^{2+}-genes and one iteration of matched random genes. (**B**) UMAP embedding based on *scHPF* of Ca^{2+}-genes calculated from a subset of the dataset from Zeisel *et al.* [14], colored by Ca^{2+}-states. (**C**) UMAP feature plots based on *scHPF* of ten different iterations of random genes, colored by Ca^{2+}-states. (**D**) LISI scores measuring neighborhood heterogeneity (lower values indicate more homogeneous neighborhoods), computed for the Ca^{2+}-based UMAP and for each of ten iterations using matched random genes. (**E**) Confusion matrix showing the true and predicted Ca^{2+}-states using the Ca^{2+} classifiers. (**F**) Confusion matrix showing the true and predicted cell types using the Ca^{2+} classifiers. (**G**, **H**) Boxplots showing the precision, recall, and F1-scores of classifiers trained on Ca^{2+}-genes or matched random genes, evaluated across Ca^{2+}-states (**G**) or cell types (**H**). For the box plots, the center line shows the median, the red cross shows the mean, the upper and lower boundaries of the box show the upper and lower quartiles, the whiskers extend to 1.5× the interquartile range, and open circles indicate outliers.

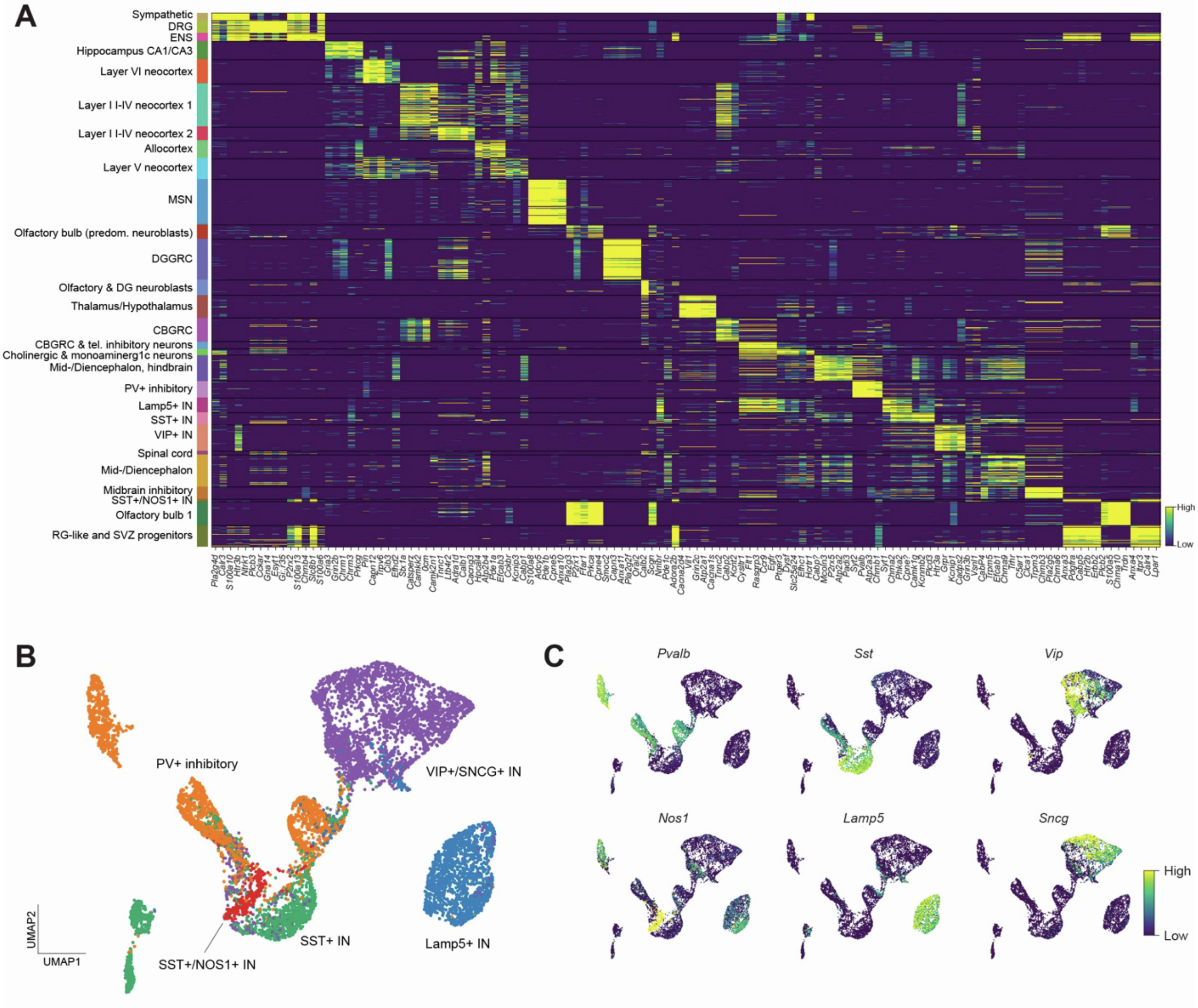

Figure S4. Gene enrichment patterns across Ca^{2+}-states. Gene enrichment patterns across Ca^{2+}-states in the Zeisel *et al.* [14] dataset. (**A**) Heatmap of cell-gene scores showing the top genes for each Ca^{2+}-state across the entire dataset. (**B**, **C**) UMAP embedding of interneurons colored by Ca^{2+}-states (**B**) and feature plots of marker genes used to identify cortical interneuron subpopulations (**C**).

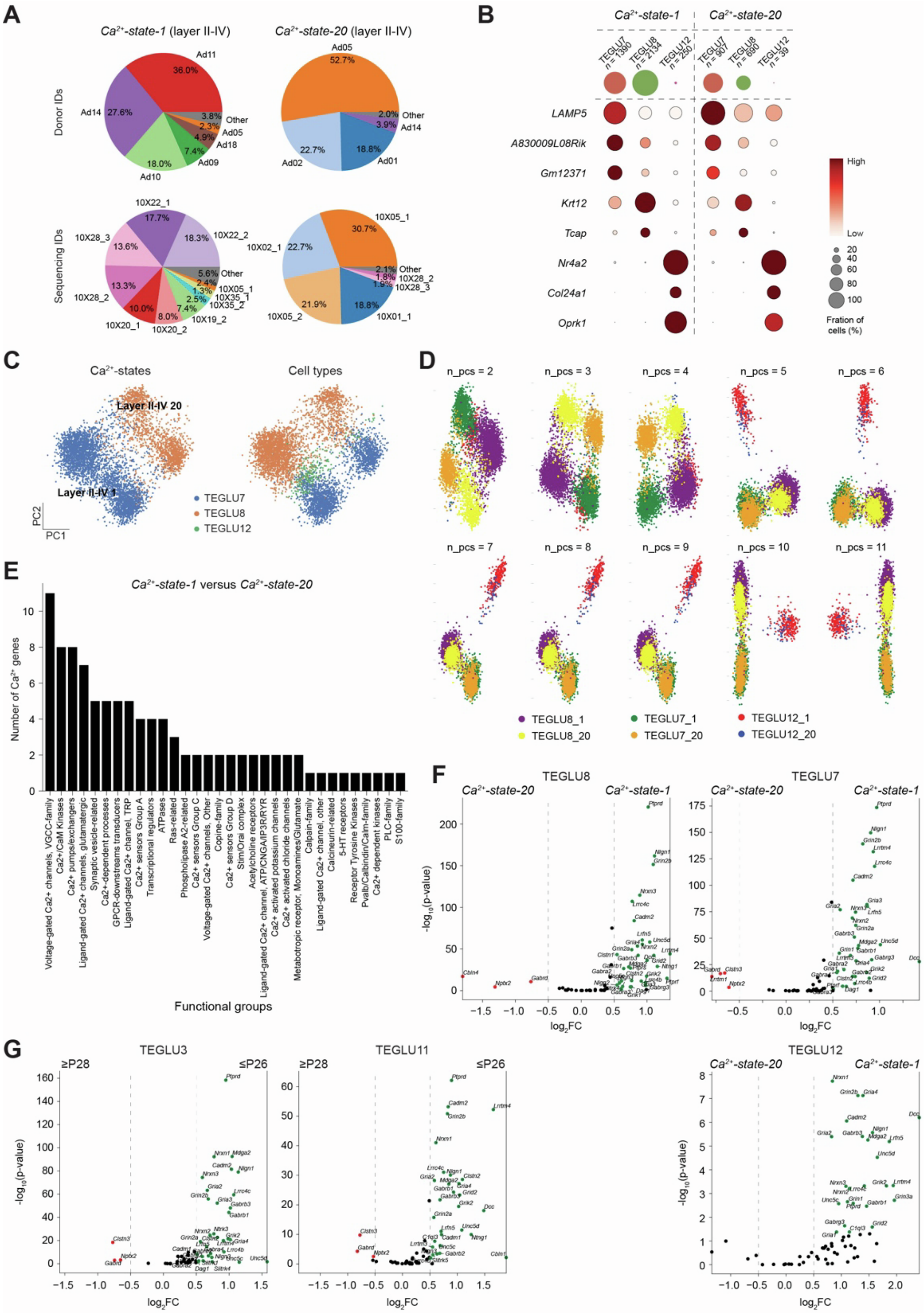

Figure S5. Age- and activity-associated differences between Ca^{2+}-states in neocortical layer II-IV neurons. Age- and activity-associated differences between Ca^{2+}-state-1 and Ca^{2+}-state-20 in neocortical layer II-IV neurons. (**A**) Pie charts showing the distribution of donor identifiers (Donor IDs; top) and sequencing experiment identifiers (Sequencing IDs; bottom) for cells in the layer II-IV *Ca^{2+}-state-1* and *Ca^{2+}-state-20*. (**B**) Dot plot showing the expression of marker genes for the three cell types present in *Ca^{2+}-state-1* and *Ca^{2+}-state-20*, demonstrating that cell type composition is shared between the two states. (**C**) Scatter plots of the first two principal components (PCs) of the global transcriptome, colored by Ca^{2+}-state (left) and cell type (right). (**D**) Scatter plots of two-dimensional Linear Discriminant Analysis (LDA) computed using incremental numbers of PCs (n_pcs), colored by cell type and Ca^{2+}-state. (**E**) Bar plot showing differentially expressed Ca^{2+}-genes between *Ca^{2+}-state-1* and *Ca^{2+}-state-20*, grouped by functional groups. (**F**) Volcano plots of synapse formation and remodeling-related genes, stratified by cell type, comparing *Ca^{2+}-state-1* and *Ca^{2+}-state-20*. (**G**) Volcano plots of synapse-related genes, stratified by cell type, comparing cells aged ≤P26 and ≥P28.

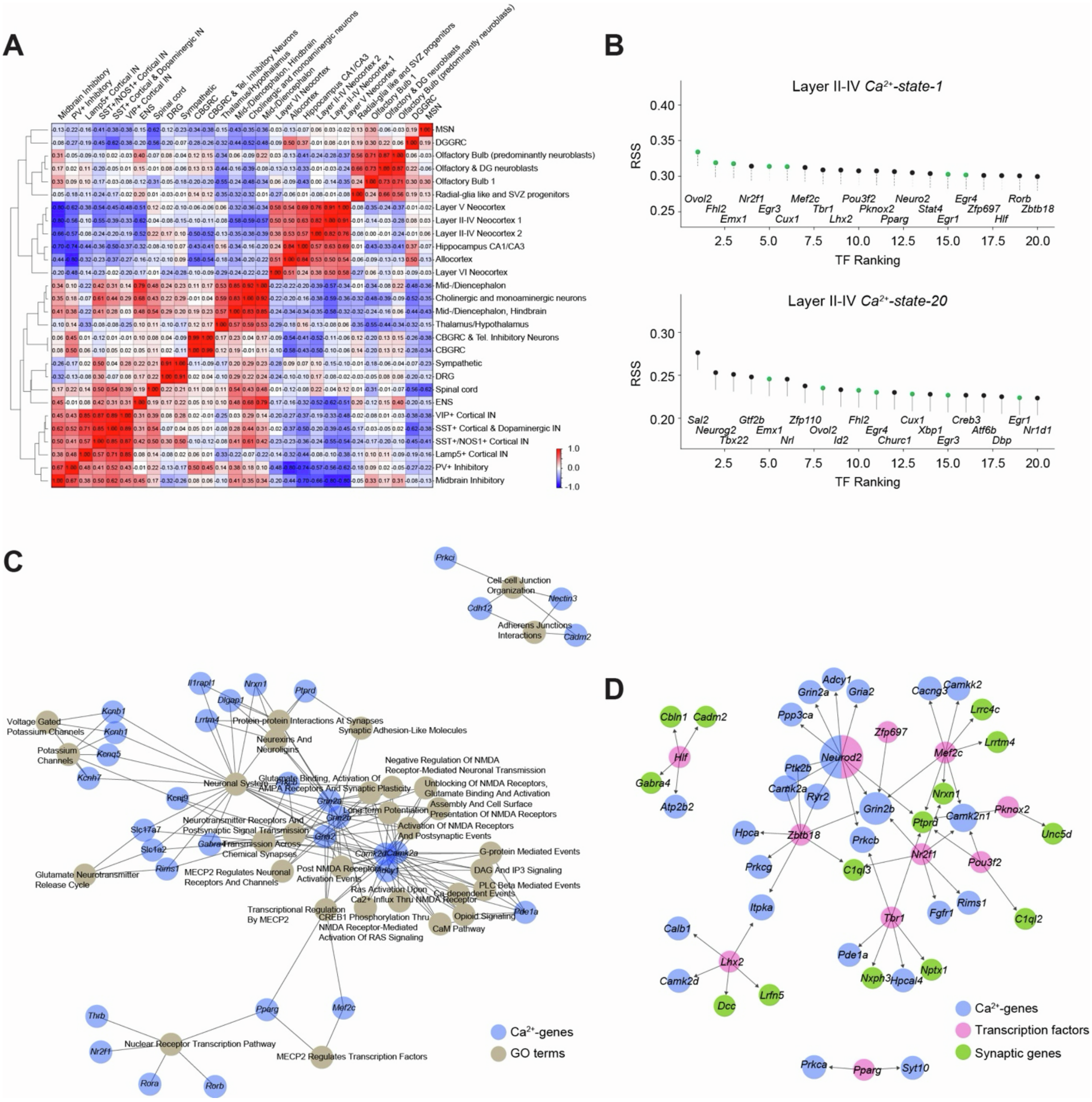

Figure S6. Gene regulatory network inference using *pySCENIC*. (**A**) Correlation matrix of predicted transcription factor (TF) activities across the entire Zeisel *et al*. [14] dataset. (**B**) Top enriched TFs for neocortical layer II-IV *Ca^{2+}-state-1* and *Ca^{2+}-state-20*, calculated using regulon specificity scores (RSS). (**C**) Networks of enriched gene ontology terms and synapse-related genes inferred from the non-shared enriched TFs in *Ca^{2+}-state-1.* (**D**) Networks of TFs enriched in *Ca^{2+}-state-1* and their predicted synapse-related gene targets and Ca^{2+}-genes.

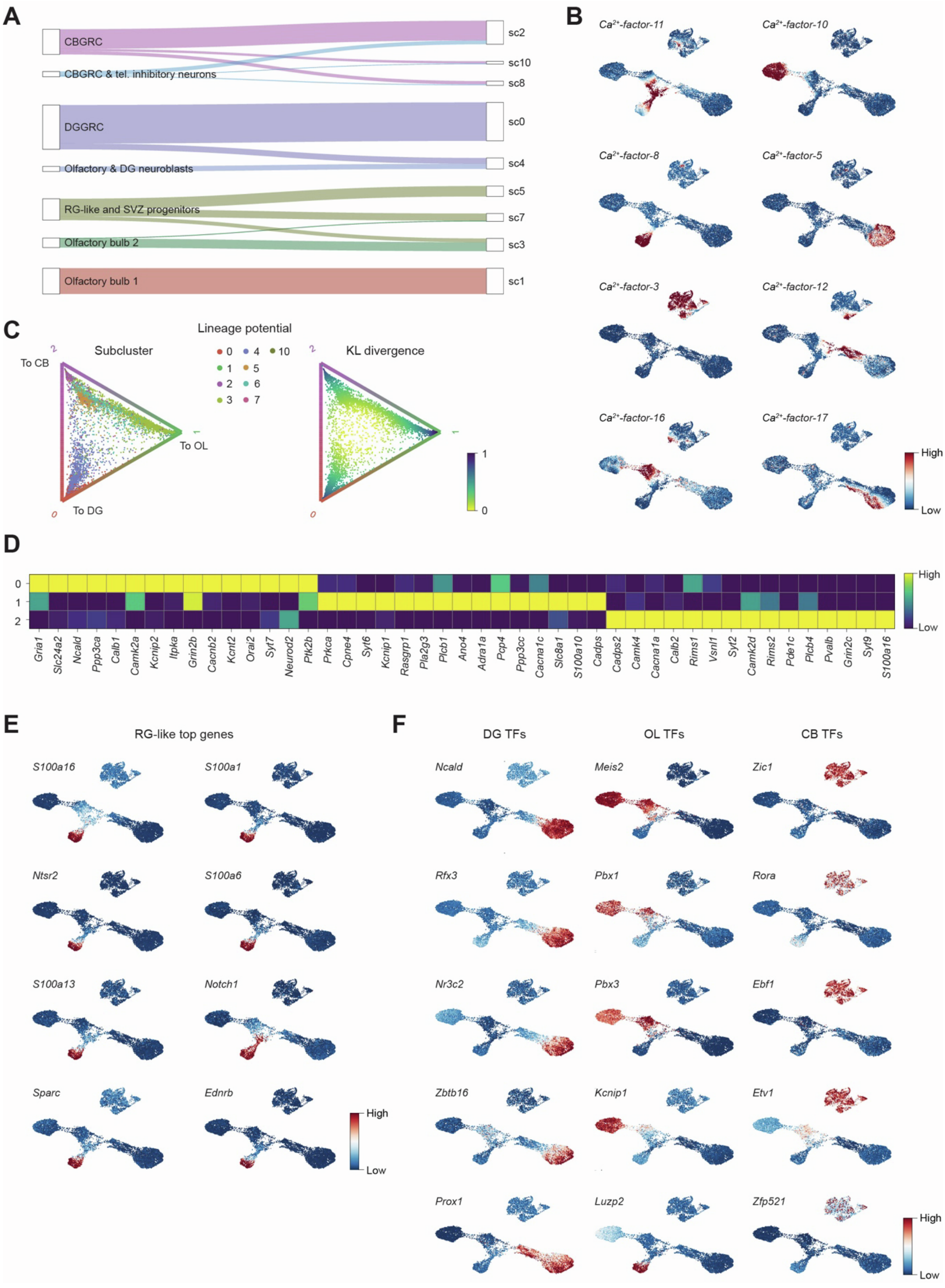

Figure S7. Differential gene and Ca^{2+}-factor enrichment across postnatal developmental trajectories. (**A**) Sankey diagram showing the subclustering of the relevant CB, DG, and OL Ca^{2+}-states. (**B**) UMAP feature plots showing differential enrichment of Ca^{2+}-factors across the developmental subset. (**C**) Circular projections of transition probabilities toward each of the three lineages, colored by subcluster (left) and Kullback–Leibler (KL) divergence (right). (**D**) Matrix plot showing Ca^{2+}-genes enriched in each of the three terminal states. (**E**) UMAP feature plots of Ca^{2+}-genes enriched in the RG-like cluster. (**F**) UMAP feature plots of TFs enriched along each of the three lineages.

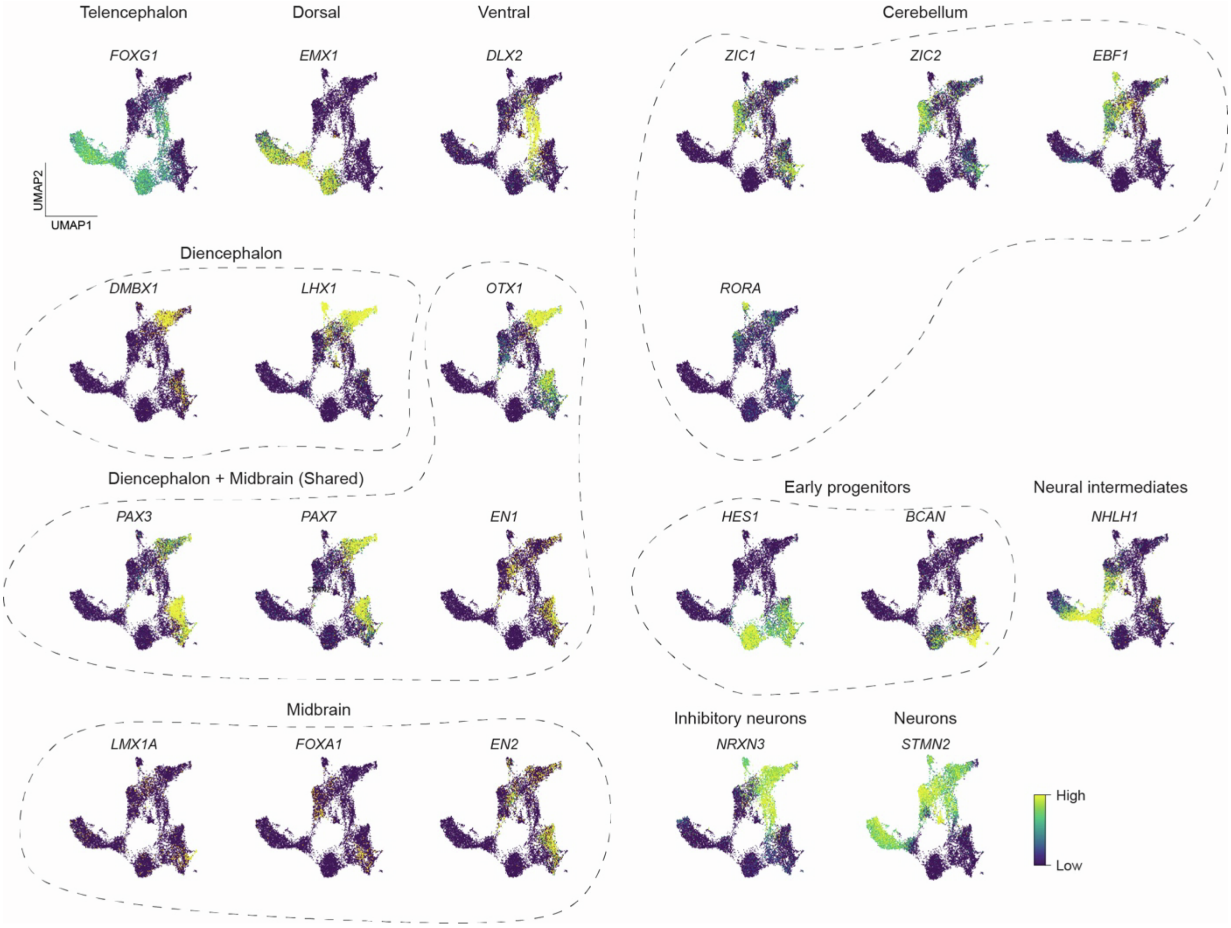

Figure S8. UMAP feature plots highlighting the genes used to re-annotate the human developmental forebrain dataset. UMAP feature plots highlighting the genes used to re-annotate the human developmental forebrain dataset from van Bruggen *et al*. [30].

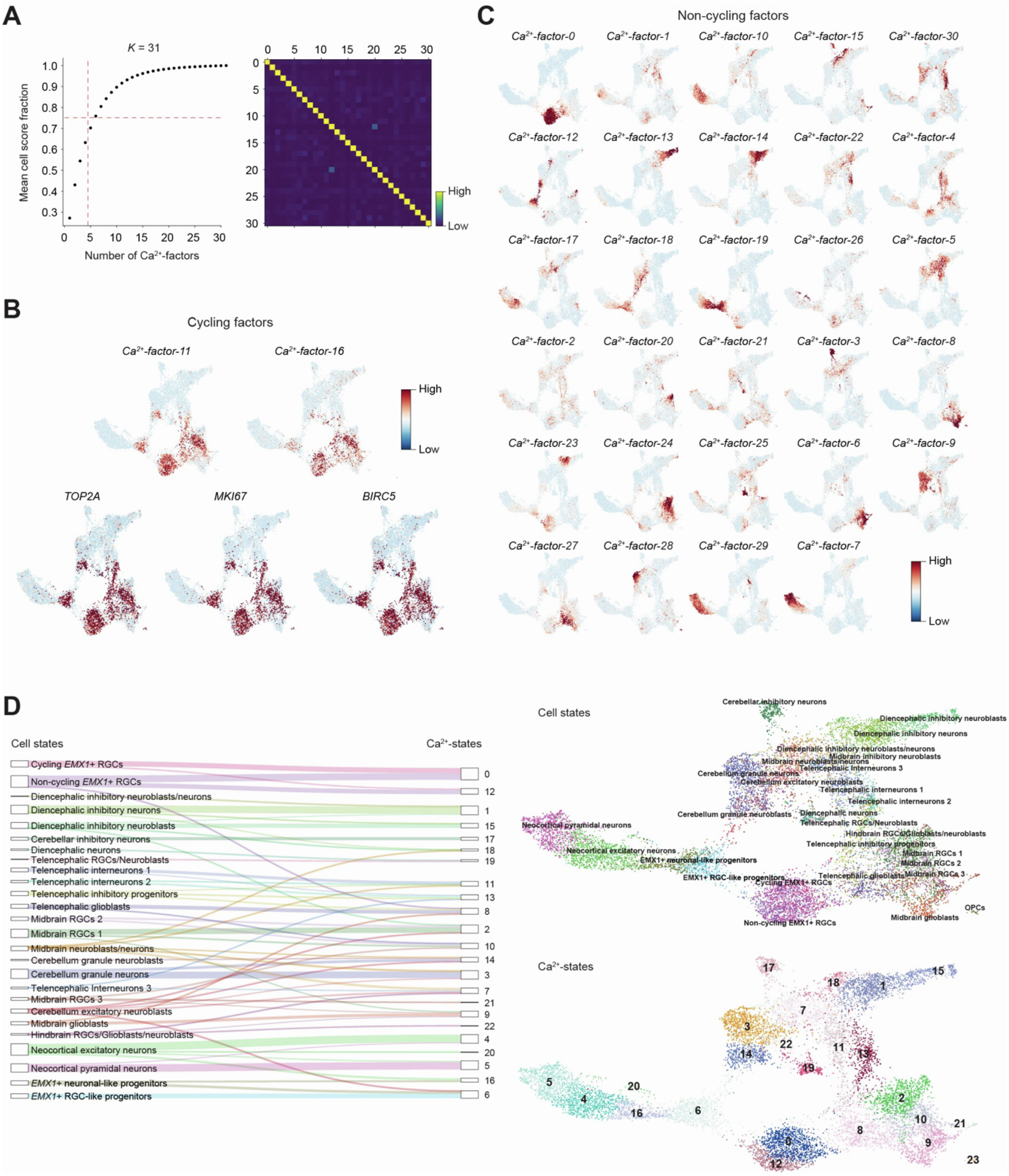

Figure S9. Clustering strategy used to analyze the van Bruggen dataset. Overview of the clustering strategy used to analyze the dataset from van Bruggen *et al.* [30]. (**A**) *Left*: Mean cell score fraction plotted against the number of Ca^{2+}-factors. As for Zeisel *et al.* [14], *scHPF* was iteratively run with *K* manually tuned to the maximum value at which four Ca^{2+}-factors captured, on average, 70% of the cell scores. *Right:* Heatmap of normalized mutual information scores between Ca^{2+}-factors. (**B**) UMAP feature plots showing the identified cycling *Ca^{2+}-factor-11* and *Ca^{2+}-factor-16* (top), along with selected cell cycle-related genes for reference (bottom). (**C**) UMAP feature plots of the 29 remaining non-cycling Ca^{2+}-factors, showing their differential enrichment across the dataset. (**D**) Relationship between cell states and Ca^{2+}-states, visualized as a Sankey diagram (left) and a UMAP embedding (right).

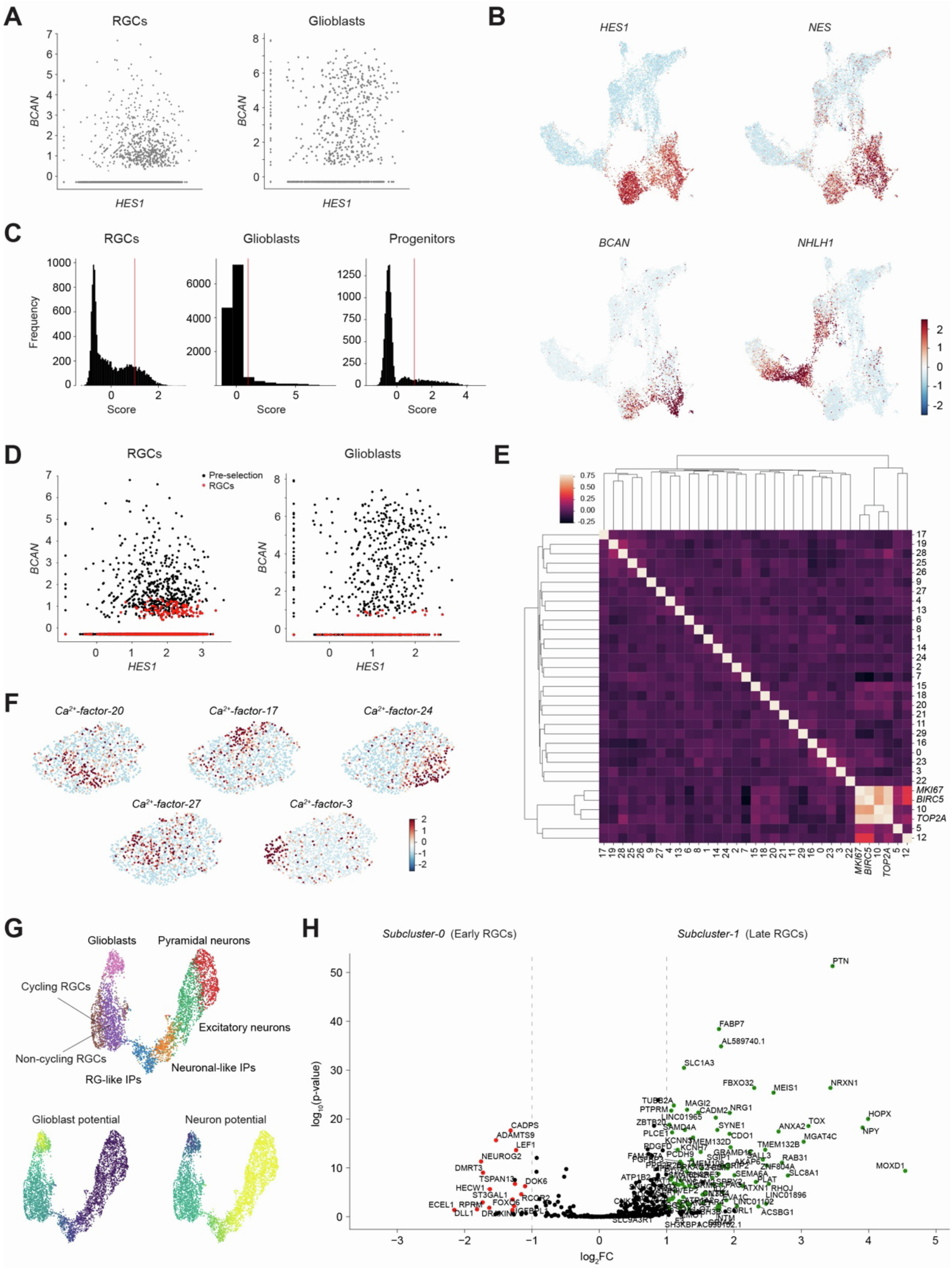

Figure S10. Radial glial cell heterogeneity during human development. **(A)** Scatter plots of *BCAN* versus *HES1* expression in clusters annotated as RGCs and glioblasts. **(B)** UMAP feature plots showing the expression of *HES1*, *NES*, *BCAN*, and *NHLH1*. **(C)** Histograms showing the distribution of RGC scores (left), glioblast scores (center), and neural progenitor scores (right). The red line indicates the threshold used for score binarization. **(D)** As in (A), with selected RGCs highlighted in red. **(E)** Correlation matrix of Ca^{2+}-factors calculated exclusively from RGCs and cycling genes. **(F)** UMAP feature plots showing the differential enrichment of Ca^{2+}-factors across the five identified subclusters within RGCs. Representative enriched Ca^{2+}-factors for each subcluster are indicated above. **(G)** UMAP feature plots showing fate probabilities of all *EMX1*+ cells toward glioblastic and neuronal linages, respectively. **(H)** Differential expression analysis comparing *Subcluster-0* (left) and *Subcluster-1* (right), highlighting genes associated with early and late radial glial states.

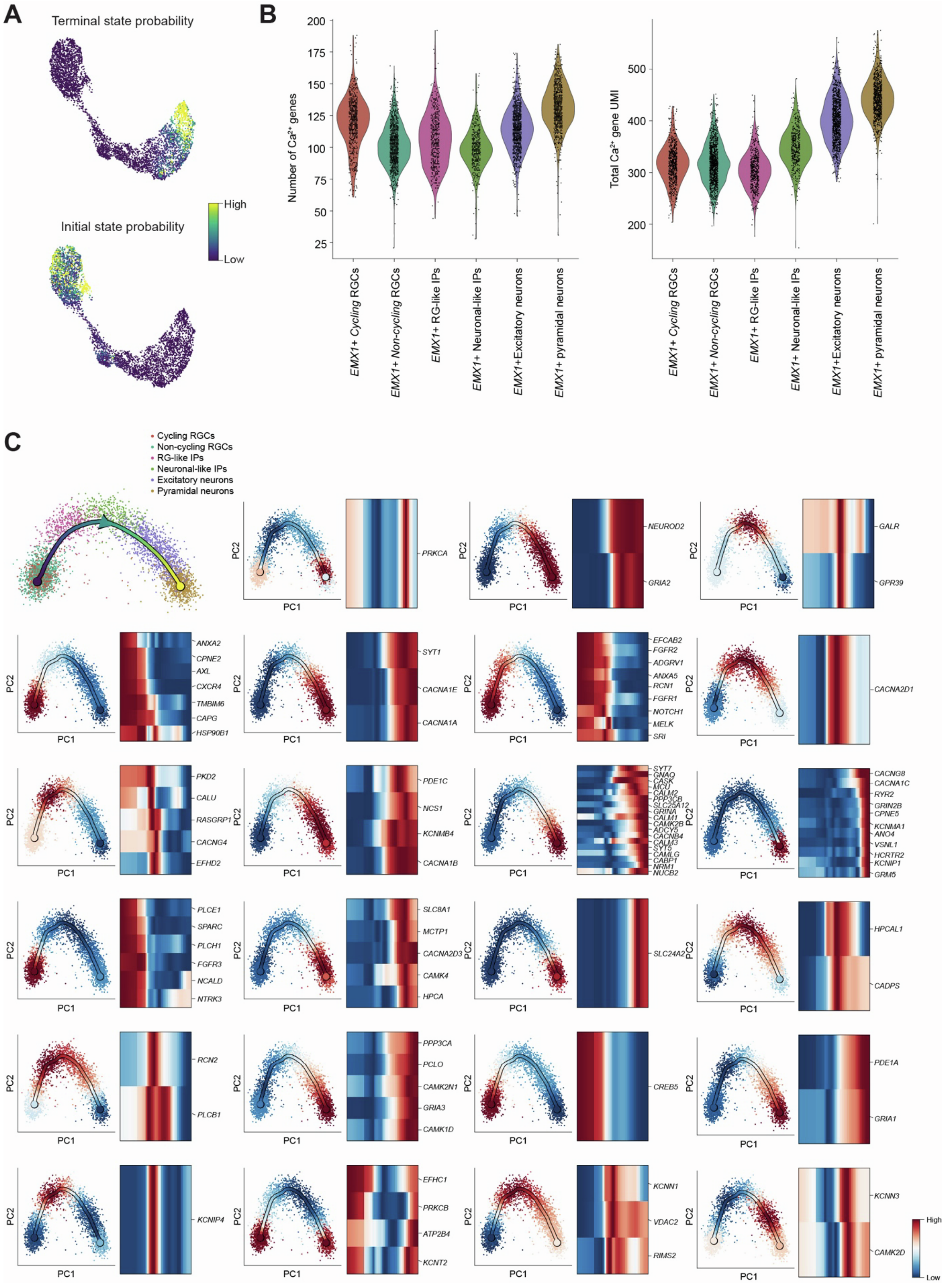

Figure S11. **Delineation of Ca^{2+} dynamics during human neocortical development**. (**A**) UMAP feature plots showing the initial and terminal state probabilities across the *EMX1+* lineage. (**B**) Violin plots showing the number of Ca^{2+}-genes (left) and UMI counts (right) per cell, grouped by cell state. (**C**) Scatter plots of PC1 versus PC2, colored by the aggregated expressions of each Ca^{2+}-gene program as computed by *scFates*.

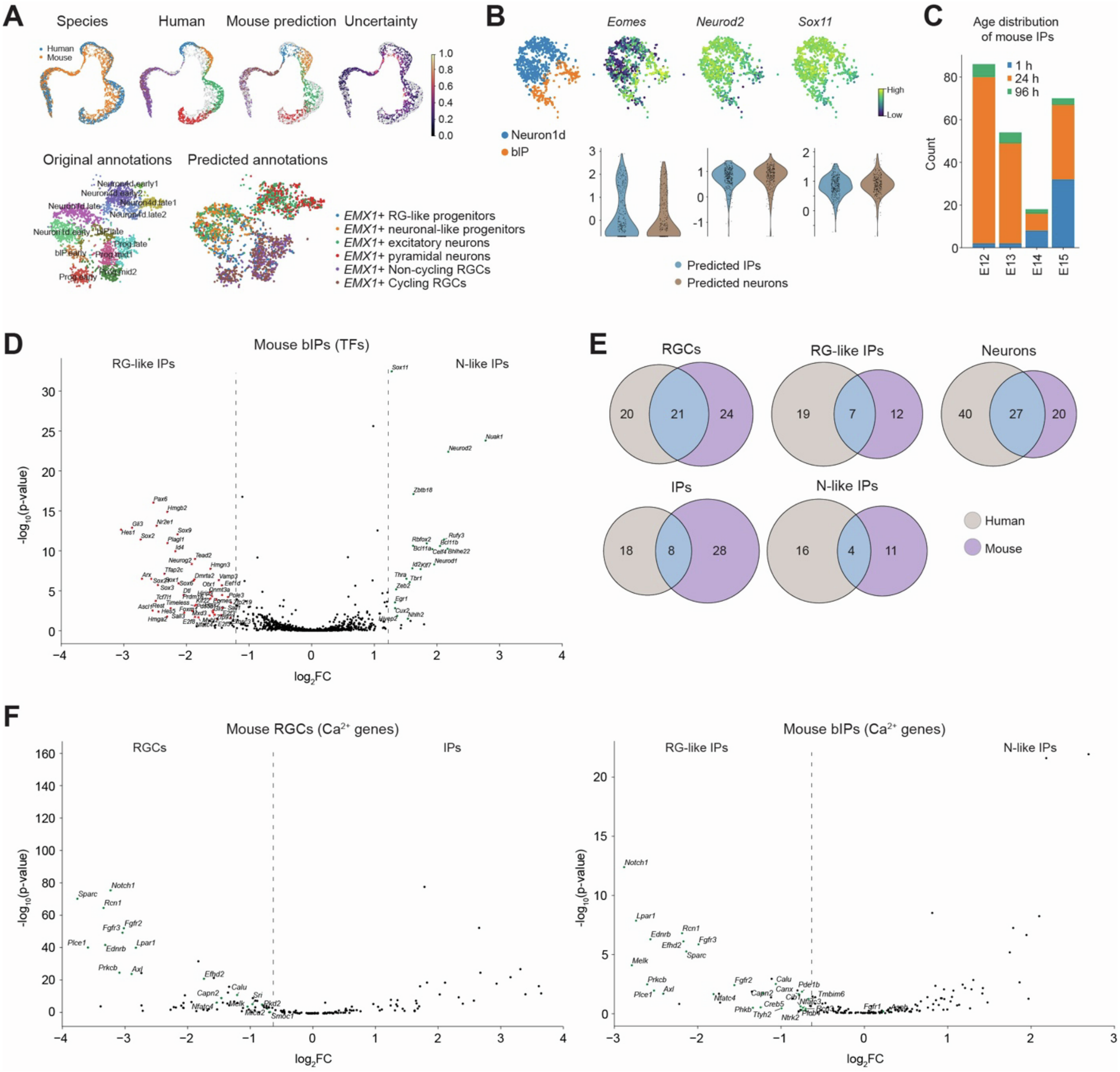

Figure S12. Cross-species comparison of Ca^{2+} dynamics during neocortical development. (**A**) *Top:* Joint embedding of human (van Bruggen *et al*. [30]) and mouse (Telley *et al*. [35]) neocortical cells, showing the distribution of human-defined cell states, transferred labels in the mouse dataset, and prediction uncertainty. *Bottom:* t-distributed Stochastic Neighbor Embedding (t-SNE) plot highlighting the original mouse annotations and the corresponding predicted human-defined annotations. (**B**) *Top:* t-SNE embedding of Neuron1d and bIP populations, as defined by the original mouse annotations, with feature plots of *Eomes, Neurod2,* and *Sox11*. *Bottom:* Violin plot of the same genes, grouped by the predicted human-defined annotations. (**C**) Stacked bar plots showing the age distributions of mouse intermediate progenitors (IPs), grouped by embryonic stage. (**D**) Volcano plot of differentially expressed TFs in RG-like IPs (red) versus neuronal-like IPs (green). (**E**) Venn diagrams showing the degree of conservation between human-defined cell states and their predicted mouse counterparts. (**F**) *Left:* Volcano plot of differentially expressed Ca^{2+}-genes in mouse RGCs (green) compared with mouse IPs. *Right:* Same analysis comparing predicted RG-like IPs and predicted neuronal-like (N-like) IPs in mouse.
